# A novel nucleolin-binding peptide for Cancer Theranostics

**DOI:** 10.7150/thno.43502

**Published:** 2020-07-14

**Authors:** Jae-Hyun Kim, Chanhyung Bae, Min-Jung Kim, In-Hye Song, Jae-Ha Ryu, Jang-Hyun Choi, Choong-Jae Lee, Jeong-Seok Nam, Jae Il Kim

**Affiliations:** 1 School of Life Sciences, Gwangju Institute of Science and Technology, Gwangju 61005, Republic of Korea.; 2 Molecular Physiology and Biophysics Section, Porter Neuroscience Research Center, National Institute of Neurological Disorders and Stroke, National Institutes of Health, Bethesda, United States.; 3 Pilot Plant, Anygen, Gwangju, Technopark, 333 Cheomdankwagi-ro, Buk-gu, Gwangju, 61008, Republic of Korea.

**Keywords:** peptide, one-bead-one-compound, multiple-antigen-peptide, nucleolin, paclitaxel

## Abstract

**Background:** Cancer-specific ligands have been of great interest as pharmaceutical carriers due to the potential for site-specific delivery. In particular, cancer-specific peptides have many advantages over nanoparticles and antibodies, including high biocompatibility, low immunogenicity, and the formation of nontoxic metabolites. The goal of the present study was the development of a novel cancer-specific ligand.

**Methods:** Cancer-specific peptide ligands were screened using a one-bead-one-compound (OBOC) combinatorial method combined with a multiple-antigen-peptide (MAP) synthesis method. The specificity of the peptide ligands toward cancer cells was tested *in vitro* using a whole-cell binding assay, flow cytometry, and fluorescence confocal microscopy. The tissue distribution profile and therapeutic efficacy of a paclitaxel (PTX)-conjugated peptide ligand was assessed *in vivo* using xenograft mouse models.

**Results:** We discovered that AGM-330 specifically bound to cancer cells *in vitro* and *in vivo*. Treatment with PTX-conjugated AGM-330 dramatically inhibited cancer cell growth *in vitro* and *in vivo* compared to treatment with PTX alone. The results of pull-down assay and LC-MS/MS analyses showed that membrane nucleolin (NCL) was the target protein of AGM-330. Although NCL is known as a nuclear protein, we observed that it was overexpressed on the membranes of cancer cells. In particular, membrane NCL neutralization inhibited growth in cancer cells *in vitro*.

**Conclusions:** In summary, our findings indicated that NCL-targeting AGM-330 has great potential for use in cancer diagnosis and targeted drug delivery in cancer therapy.

## Introduction

The use of cancer-specific ligands as pharmaceutical carriers allows relatively site-specific delivery of a higher payload of chemotherapeutic agents than that which is achieved by conventional drugs, which in turn decreases systemic toxicity 1. Among the cancer-specific ligands, nanoparticles and antibodies have been widely studied in clinical cancer diagnosis and therapy 2,3. Theragnostic nanoparticles and antibodies show great promise in the emerging field of personalized medicine because they allow the detection and monitoring of an individual patient's cancer at an early stage and the delivery of anticancer agents over an extended period for enhanced therapeutic efficacy 4,5. Although nanoparticles are promising drug carrier systems, their instability in circulation, inadequate tissue distribution, and cytotoxicity present unresolved limitations to practical applications 6,7. Additionally, the limitations of therapeutic antibodies include slow delivery and diffusion into tumor tissue due to their large size 8.

As an alternative to classical diagnostic and therapeutic methods, cancer-specific peptides can be used to increase treatment efficiency and reduce the side effects associated with nanoparticle and antibody cancer therapy 9. Peptide ligands have many advantages, including easy synthesis in large quantities, low immunogenicity, the generation of nontoxic metabolites, and high *in vivo* biocompatibility 10. Among the many ways to discover peptides, one-bead-one-compound (OBOC) combinatorial methods are one of the powerful tools for screening peptide ligands 11,12. Interestingly, peptide screening approaches based on OBOC combinatorial libraries have facilitated the discovery of novel peptide ligands for cellular targeting in cancer and other diseases 13-16. Although numerous cancer-specific peptides have been isolated using *in vitro* OBOC combinatorial screening or other methods, several challenges remain. In particular, the primary drawback in the use of a peptide as a drug is its extremely short half-life due to very rapid cleavage by different peptidases 17. We aimed to overcome the problems outlined above by developing a dedicated approach that synthesizes bioactive peptides in multiple-antigen peptide (MAP) dendrimeric form. The synthesis of monomeric peptides in dendrimeric forms can result in increased stability due to acquired resistance to protease and peptidase activity 18-20.

In this study, we combined OBOC combinatorial screening and MAP synthesis and discovered a peptide ligand (AGM-330) that specifically binds to human breast and colorectal cancer cells. *In vivo* fluorescence imaging demonstrated that AGM-330 was specifically distributed more in tumors than in normal tissues. Additionally, treatment with paclitaxel (PTX)-conjugated AGM-330 improved paclitaxel accumulation in cancer cells and inhibited breast and colorectal cancer cells more efficiently than treatment with PTX alone *in vitro*. Furthermore, treatment with PTX-conjugated AGM-330 facilitates the specific localization of PTX in tumor tissues, resulting in increased cytotoxicity, enhanced drug accumulation, and improved anticancer efficacy in a xenograft model of breast cancer. Moreover, pull-down assays followed by LC-MS/MS indicated that nucleolin (NCL) is the target protein of AGM-330. NCL is the most abundant protein in the nucleolus. However, in this study, we observed overexpression of NCL in the cancer cell membrane, and neutralization of cell membrane NCL by anti-NCL antibodies inhibited cancer cell proliferation and increased apoptotic rates *in vitro*. Because of its carcinogenic roles and expression on cancer cell membranes, NCL represents an attractive target for cancer treatments. Taken together, these results indicated that AGM-330 is a novel tumor-targeting peptide for cancer diagnosis and therapy.

## Methods

### Synthesis of the initial and refined OBOC libraries

OBOC libraries were synthesized on solid-phase TentaGel MB NH_2_ resin (Rapp Polymere GmbH, Tübingen, Germany). A “split-mix” synthesis method was performed to construct the combinatorial OBOC libraries, each containing random libraries of millions of beads/ligands. Five grams of Tentagel MB NH_2_ resin (200 μm, 520,000 beads/g) was used for the synthesis of approximately 2,600,000 OBOC libraries. The ligand on the bead surface was synthesized by standard solid-phase peptide synthesis techniques using 9-fluorenylmethoxycarbonyl (Fmoc) chemistry and N-hydroxybenzotriazole (HOBt; *GL Biochem*, Shanghai, China)/N,N'-diisopropylcarbodiimide (DIC; GL Biochem) coupling. The completion of coupling was confirmed with a ninhydrin test. The beads were stored in 70% ethanol at 4 °C until use.

### Ethics and cell culture

All work related to human tissues was preapproved by the Institutional Review Board (IRB) at the Gwangju Institute of Science and Technology (#20191008-BR-48-03-02). All animal experiments were performed according to the Institutional Animal Care and Use Committee (IACUC) of the Gwangju Institute of Science and Technology (GIST-2019-040). All cultures were grown in a humidified incubator maintained at 37 °C under an atmosphere with 95% air/5% CO_2_. MCF-10A normal human breast cell lines were obtained from the American Type Culture Collection and propagated in MEGM complete growth medium (MEGM, Lonza, Walkersville, MD) supplemented with bovine pituitary extract (Cambrex Bioscience, Walkersville, MD). The normal human colorectal cell line CCD-18Co; human breast and colorectal cancer cell lines, including MCF-7, MDA-MB-231, HT-29, and HCT-116; and Jurkat T cells were obtained from the Korea Cell Line Bank (Seoul, Korea). The luciferase-expressing cancer cell line MDA-MB-231 was obtained from PerkinElmer (PerkinElmer, Hopkinton, MA). Each cancer cell line was grown in RPMI1640 (Gibco, Waltham, USA) and DMEM (Gibco) supplemented with 10% heat-inactivated fetal bovine serum (FBS; Gibco), 0.1 mg/ml streptomycin (Gibco), and 100 units/ml penicillin (Gibco).

### Screening of the OBOC library for cancer-specific ligands

The beads were washed extensively with double-distilled water and phosphate-buffered saline (PBS; Welgene Inc., Korea) before screening. Cancer and normal cells were detached from culture dishes with trypsin/EDTA (Gibco), washed with their corresponding culture medium, resuspended at 10^6^ cells/ml, and incubated with OBOC beads in Petri dishes in a humidified CO_2_ incubator at 37 °C with shaking (60 rpm). Beads bound by cells appeared under a microscope as rosettes in which a central bead was covered by one or more layers of cells. The positive beads were picked with a pipette under an inverted microscope, treated with guanidine-HCL (8 M, 20 min) to remove cells and proteins on the bead surface, and subjected to a second round of screening with normal breast epithelial cells to eliminate false positive binding. Only beads exhibiting cell binding at both rounds were sent for peptide sequencing.

### Chemical synthesis of peptides and 2'-maleimide-PTX

All peptides and 2'-maleimide-PTX were synthesized by AnyGen (Gwangju, Korea) using solid-phase peptide synthesis and Steglich esterification, respectively. The purity and molecular masses of the peptides and 2'-maleimide-PTX were determined using HPLC and matrix-assisted laser desorption ionization time-of-flight mass spectrometry (MALDI-TOF MS), respectively (Shimadzu, Kyoto, Japan).

### Synthesis of AGM-330-PTX

AGM-330 and thiol intermediates were dissolved in 500 μM dimethylformamide (DMF; Duksan Chemical, Korea) and mixed with a solution of Alexa680-C_2_-maleimide (Thermo Fisher Scientific, Rockford, IL), FITC-5-maleimide (Thermo Fisher Scientific), and 2'-maleimide-PTX in DMF (5 mM; 10 equivalents) and 0.1% v/v N,N-diisopropylethylamine (DIPEA; Duksan). The mixture was stirred at room temperature, and the reaction was monitored by HPLC. After the reaction, the product was purified by HPLC (Shimadzu), and the molecular weight was subsequently characterized using MALDI-TOF MS (Shimadzu).

### *In vitro* stability test

Stock solutions of peptides (100 μM) were diluted by a factor of 10 with pre-warmed 100% human serum (Sigma-Aldrich, St. Louis, MO, USA) and incubated at 37 °C for 0, 3, 6, 9, and 24 h. Controls with peptides in PBS were included. The action was stopped by denaturing the serum proteins with urea at a final concentration of 3 M at 4 °C for 10 min, followed by precipitation of serum proteins with trichloroacetic acid at a final concentration of 7% (v/v) (4 °C, 10 min) and centrifugation (17,000 × g, 10 min). The supernatant of each sample was recovered and run on an analytical column using a linear gradient of 5-65% solvent B for 30 min at a flow rate of 1 ml/min, where solvent A was water containing 0.1% TFA and solvent B was acetonitrile containing 0.1% TFA. The percentage of peptide remaining in serum-treated samples was determined by comparing the height of the peptide peak obtained at each time point with that of the peptide peak obtained at the 0 time point. Each experiment was performed in triplicate.

### *In vivo* stability test

Mice were administrated equivalent amounts of AGM-330 m, AGM-330d and AGM-330 in a single intravenous injection. Blood samples were collected at 0, 0.167, 0.5, 1, 2, 4, 8, 12 and 24 h from one eye using heparinized capillary tubes (DWK life sciences, Mainz, Germany) that were immediately chilled on ice. After 15 min of centrifugation at 5,000 rpm and 4 °C, the plasma was obtained and stored at -80 °C. Plasma peptide concentrations were quantified by ELISA, and pharmacokinetic profiles were analyzed using Phoenix WinNonlin 8.1 (*Pharsight* Corporation, Mountain View, CA, USA). The area under the plasma concentration-time curve (AUC), distribution half-time (T_1/2_), maximum plasma concentration (C_max_) and time required to reach maximum plasma concentration (T_max_) values were directly determined from the experimental data.

### Flow cytometry analysis

Fluorescence-activated cell-sorting (FACS) analysis was used to check the binding of the FITC labeled peptides (FITC-peptide). The stock solution for the FITC-peptides (100 μM) was prepared by dissolving the peptide in PBS. MCF-10A, MCF-7, MDA-MB-231, CCD-18Co, HT-29, and HCT-116 cells were seeded in 6-well plates (10^5^ cells/well) containing 3 ml of medium, and the plates were then incubated at 37 °C overnight. The following day, the media was replaced with fresh FBS-free medium (1 ml) containing FITC-peptide (1 μM) and further incubated at 37 °C for 30 min. Thereafter, the medium was removed, and the cells were washed with cold PBS to remove any remaining peptide. Adequate trypsin/EDTA was added to each well, which was followed by a 3 ~ 5 min incubation at 37 °C. Trypsin/EDTA was immediately neutralized by adding medium, and the cell suspension was then transferred into centrifuge tubes. The cells were separated and washed twice with PBS. The control cells were treated similarly but without peptide. The prepared cells were analyzed on a FACScanto II (BD Biosciences, San Jose, CA, USA) flow cytometer, and the FACS data were analyzed using FlowJo (TreeStar, Ashland, OR, USA).

### Biotin pull-down assay

The cell lysates (500 μg/ml) were incubated with biotinylated AGM-330 for 12 h at 4 °C and then used in pull-down assays with streptavidin beads (Thermo Fisher Scientific) for 1 h at room temperature. After the incubation, the beads were washed three times with washing buffer. Then, elution buffer was added to the beads, and the extracted proteins were analyzed by SDS-PAGE. To identify and characterize the proteins derived from the biotin pull-down assay, LC-coupled ESI-MS/MS analysis was performed at ProteomeTech (Seoul, Korea).

### AGM-330 binding domain identification

The biotin pull-down assay was performed as previously described. The NCL constructs, including GFP-NCL (residues 1-710), GFP-ΔN-NCL (residues 322-710), and GFP-ΔC-NCL (residues 1-321) were generated from the human NCL cDNA clone (Addgene, Cambridge, MA, USA) and subcloned into the *Xho*I and *Bam*HI sites of the vector pEGFP-C2 (Addgene). These vectors were transfected into cells using Lipofectamine 2000 (Invitrogen, Carlsbad, CA, USA) according to the manufacturer's recommendations. Biotinylated AGM-330 peptides were added to the streptavidin beads (Thermo Fisher Scientific), and the mixture was incubated for 1 h at room temperature with shaking. Subsequently, the beads were washed 3 times with wash buffer before being added to 300 μl of lysate prepared from transfected cells containing GFP-tagged NCL proteins. The reaction mixture was incubated for 12 h at 4 °C to allow binding between AGM-330 and GFP-tagged NCL proteins. The beads were subsequently washed with wash buffer. Then, an equal volume of 2× electrophoresis sample buffer was added to the beads, and proteins were extracted from the beads by heating at 95 °C for 5 min before being analyzed by SDS-PAGE and immunoblot analysis.

### Surface plasmon resonance

The surface plasmon resonance (SPR) analyses were performed at 25 °C on a Biacore T-200 instrument (GE Healthcare, Freiburg, Germany) equipped with research-grade CM5 sensor chips (GE Healthcare). The running buffer was HBS-EP [10 mM HEPES (pH 7.4), 0.15 M NaCl, 3.4 mM EDTA, and 0.05% Triton-X 100]. Coupling reagents, N-hydroxysuccinimide, 1-ethyl-3-(3-dimethylaminopropyl)-carbodiimide hydrochloride, ethanolamine hydrochloride, and HBS-EP running buffer were purchased from Biacore. To measure the binding properties of AGM-330 to NCL (OriGene, Rockville, MD), full length recombinant NCL was immobilized onto the surface of a sensor chip CM5 using the standard amine coupling chemistry. Typically, 1000 RU of recombinant NCL was immobilized onto the sensor surface. Binding curves were recorded by injecting AGM-330 (20 nM - 2.5 μM) over the immobilized NCL at a constant flow rate of 30 μl/min. Association and dissociation phases were recorded for 180 and 300 s, respectively. The rate constants of the interactions described above were calculated by nonlinear analysis of the association and dissociation curves using the SPR kinetic evaluation software BIAevaluation (GE Healthcare). The equilibrium dissociation constants (*K*_D_) values were calculated from the values of the association rate constant *k*_a_ and dissociation rate constant *k*_d_ values according to the thermodynamic relationship *K*_D_ = *k*_d_/*k*_a_.

### Protein isolation and Western blot analysis

Cells were lysed in RIPA buffer [20 mM Tris-HCl (pH 7.5), 200 mM NaCl, 0.1% SDS, 0.1% deoxycholate, and 0.5% Triton X-100] supplemented with a protease inhibitor cocktail (Millipore). The protein concentration was measured using a Protein Assay Kit (Bio-Rad) following the manufacturer's protocol. Total protein was subjected to SDS-PAGE and transferred to a polyvinylidene difluoride membrane. The blot was probed with primary antibodies against NCL (Abcam, Cambridge, MA, USA) and GFP (Abcam). As a loading control, anti-β-actin antibody (Santa Cruz Biotechnology), anti-P-cadherin (Abcam), and anti-lamin A/C (Abcam) were used. Subsequently, the blots were washed in TBST (10 mM Tris-HCl, 50 mM NaCl, and 0.25% Tween-20) and incubated with a horseradish peroxidase-conjugated secondary antibody. The presence of target proteins was detected using enhanced chemiluminescence reagents (Thermo Fisher Scientific).

### Subcellular fractionation

Subcellular fractions of cells comprising cytosolic, nuclear, and cell membrane extracts were prepared as follows. The cells (2 × 10^6^) were lysed in 1 ml ice-cold hypotonic buffer [10 mM HEPES (pH 7.9); 150 mM NaCl; protease inhibitors, including 0.5 mM phenylmethylsulfonyl fluoride and 10 μg/ml each of aprotinin, pepstatin, and leupeptin; and 0.5% Triton X-100]. The homogenate was centrifuged to separate the nuclei and cytoplasm, membrane and mitochondria at 3,000 rpm for 5 min. Nuclei pellet was re-suspended in TBS with 0.1% SDS. The supernatant was centrifuged at 8,000 rpm for 5 min. The pellet contains mitochondria and supernatant contains cytoplasm and membrane fraction. To obtain mitochondria lysate, pellet was re-suspended in TBS with 0.1% SDS. Next, the supernatant was centrifuged at 40,000 rpm for 30 min. The supernatant contains cytoplasm fraction. The membrane fraction, obtained as the pellet, was dissolved in 200 μl ice-cold hypotonic buffer containing 1% Triton X-100 for 1 h. These samples were then analyzed by Western blot and pull-down analyses.

### Enzyme treatment

To obtained *O*-glycan cleaved NCL, the cells (2 × 10^6^) were lysed in 1 ml ice-cold hypotonic buffer [10 mM HEPES (pH 7.9); 150 mM NaCl; protease inhibitors, including 0.5 mM phenylmethylsulfonyl fluoride and 10 μg/ml each of aprotinin, pepstatin, leupeptin; and 0.5% Triton X-100]. The homogenate was centrifuged at 40,000 rpm for 30 min, after which the pelleted membrane fraction was dissolved in 200 μl of ice-cold hypotonic buffer containing 1% NP-40 for 1 h. The membrane fraction was then incubated with *O*-glycosidase (New England Biolabs, Beverly, MA, USA) and α2-3,6,8,9 neuraminidase A (New England Biolab) at 37 °C for 12 h. Subsequently, the samples were then analyzed by Western blot and pull-down analyses.

### Immunofluorescence staining

MDA-MB-231-luc xenograft tissues were formalin-fixed and paraffin-embedded for immunofluorescence staining. Cells were seeded on poly-L-lysine- and collagen I-coated cover glasses and fixed with 4% formalin. Tissues and cells were permeabilized with 0.1% Triton X-100 and blocked with 2% BSA (Sigma-Aldrich). Staining was performed as described previously using primary anti-NCL (1:200), anti-TUNEL (1:200), and anti-Ki-67 (1:500) antibodies. All nuclei were counterstained with DAPI, and immunofluorescence images were matched with H&E-stained images.

### Small interfering RNA-mediated knockdown

siRNAs targeting NCL (NM_005381.3 in NCBI database) and scramble siRNA (scr) were purchased from Bioneer (Daejeon, Korea). To promote efficient transfection, siRNAs were transfected using Lipofectamine 2000 (Invitrogen) according to the manufacturer's protocol. Three different siRNA sequences were used, and their efficiencies were evaluated by real-time PCR. The sequences of the siRNAs targeting NCL were as follows: siNCL1 sequence #1: 5'-GAGCUAACCCUUAUCUGUA(dTdT)-3' (sense) and 5'-UACAGAUAAGGGUUAGCUC(dTd T)-3' (antisense); siNCL2 sequence #2: 5'-CACAAGGAAAGAAGACGAA(dTdT)-3' (sense) and 5'-UUCGUCUUCUUUCCUUGUG(dTdT)-3' (antisense); and siNCL3 sequence #3: 5'-GAC GAAGUUUGAAUAGCUU(dTdT)-3' (sense) and 5'-AAGCUAUUCAAACUUCGUC(dTdT)-3' (antisense). The most effective siRNA was chosen based on mRNA levels determined by RT-qPCR analysis, and the resulting protein levels were validated by Western blot analysis.

### Real-time PCR

Total RNA was extracted using RNAiso (Takara, Shiga, Japan), and the RNA purity was verified by determining the 260/280 absorbance ratio. First-strand cDNA was synthesized using a PrimeScript^TM^ 1^st^ strand cDNA Synthesis kit (Takara, Shiga, Japan), and one-tenth of the cDNA was used in each PCR mixture with Power SYBR® Green PCR Master Mix (Applied Biosystems, Foster City, CA, USA). Real-time PCR was performed using a StepOnePlus Real-Time PCR System (Applied Biosystems). The relative mRNA expression of selected genes was normalized to that of β-actin and quantified using the ddCt method.

### Antibody neutralization assay

Cancer and normal cell lines were seeded in 96-well plates (1 × 10^4^ cells/well) containing 100 μl of medium, and the plates were incubated at 37 °C overnight. After incubating overnight, the cells were incubated for 24 h in the absence or presence of an anti-NCL antibody (Cell Signaling Technology, Beverly, MA, USA) at 37 °C overnight. Subsequently, cell viability was assessed by a CellVia WST-1 assay (Young in Frontier, Seoul, Korea) according to the manufacturer's instructions.

### Immunoblot analysis

Cells were washed with PBS and lysed on ice for 30 min. Protein extracts were mixed with loading buffer, and boiled at 100 °C for 5 min. A total volume of 50 µl of the denatured protein samples were loaded on 10% gels. A voltage of 80 V was applied to samples and then adjusted to 120 V when protein reached the separation gel. Next, protein was transferred to PVDF membranes for 2 h at 4 °C. PVDF membranes were washed with TBS-T 3 times, and blocked with skim milk for 1 h at room temperature. Primary antibodies were used to detect AKT (1:1000; Abcam), p-AKT (1:1000; Abcam), PI3K (1:1000; Abcam), p-PI3K (1:1000; Abcam). Membranes were treated with primary antibody overnight at 4 °C, washed with TBS-T, and incubated with secondary antibody for 1 h at room temperature. Membranes were washed and developed.

### Apoptosis assay (Annexin V)

A quantitative assessment of apoptotic cells was performed using an Annexin V-fluorescein isothiocyanate (FITC) Apoptosis Detection Kit I (BD Biosciences). Cells were collected and washed twice with cold PBS, after which they were resuspended in binding buffer (1 × 10^6^ cells/ml). Next, 100 μl of the suspension was transferred to a tube and mixed with 5 μl of FITC Annexin V and propidium iodide (PI). Then, the mixture was incubated at room temperature for 15 min in the dark after gentle vortexing. After incubation, 400 μl of 1× binding buffer was added, and the cells were analyzed by flow cytometry.

### Cell proliferation assay

Cancer and normal cells (1 × 10^4^ cells/well) were seeded in 96-well plates, incubated for 24 h, and then treated with increasing concentrations of AGM-330, PTX, and AGM-330-PTX for 48 h. Cell viability was assessed by the CellVia WST-1 assay (Young In Frontier) according to the manufacturer's instructions. The numbers of viable cells were measured at a wavelength of 450 nm using a VersaMax ELISA plate reader (Molecular Devices, San Jose, CA, USA).

### Release of PTX *in vitro*

Stock solutions of AGM-330-PTX (100 μM) were diluted 10-fold with pre-warmed 100% human serum (Sigma-Aldrich) or PBS and incubated at 37 °C for 0, 1, 2, 6 and 12 h. The reaction was stopped by denaturing the serum proteins with urea at a final concentration of 3 M at 4 °C for 10 min, which was followed by the precipitation of serum proteins with trichloroacetic acid at a final concentration of 7% (v/v; 4 °C, 10 min) and centrifugation (17,000 × g, 10 min). The supernatant of each sample was recovered and run on an analytical column using a linear gradient of 5-65% solvent B for 30 min at a flow rate of 1 ml/min, where solvent A was water containing 0.1% TFA and solvent B was acetonitrile containing 0.1% TFA. Each experiment was performed in triplicate.

### PTX uptake assay

Maleimide-PTX-Rh was synthesized by Sundia (Shanghai, China). The MDA-MB-231 cells were incubated with AGM-330-PTX-Rh (5 μmol/l) for 0.5, 1 and 2 h. Subsequently, cells were washed with PBS and fixed with 4% paraformaldehyde. Fixed cells were incubated with the antibodies against AGM-330 (Young in Frontier). The nuclei were counterstained with DAPI. MERGE represents a merged image of AGM-330, PTX-Rh, and nuclear staining by DAPI.

### Tumorigenesis experiment

All animal experiments were performed in accordance with IACUC guidelines (GIST-2019-040). For tumorigenesis experiments, anesthetized 6-week-old female NOD-scid Il2rg**^-^**^/^**^-^** mice (NPG^TM^, VITALSTAR) were inoculated in the mammary fat pad with 1 × 10^6^ MDA-MB-231-luc cells in a 100 μl volume (*n* = 5 for each group). After tumor cell inoculation, when the tumor volume reached approximately 100 mm^3^, the mice were randomly divided into the following five groups: (i) control, (ii) low-dose paclitaxel (2 mg/kg), (iii) high-dose paclitaxel (10 mg/kg), (iv) AGM-330 (16.68 mg/kg, molar equivalent to 2 mg/kg of PTX), and (v) AGM-330-PTX (19.05 mg/kg, molar equivalent to 2 mg/kg of PTX). The tumor size was measured twice a week, and the tumor volume was calculated with the following formula: volume (mm^3^) = [length (mm)] × [width (mm)]^2^ × 0.5. For the bioluminescent imaging experiment, D-luciferin (150 mg/kg; PerkinElmer) was intraperitoneally injected into each mouse, and an IVIS 100 imaging system (Xenogen, Corporation, Alameda, CA) was used for bioluminescent monitoring. Mice were anesthetized with vaporized isofurane (BK Pharm, Ilsan, Korea) and placed in an imaging chamber. After 10 min, each animal was imaged with an exposure time of 1 min. All bioluminescent image data were obtained using Living Image software (version 4.5.2, PerkinElmer). Photons detected from tumors were converted to average radiance values (photon/sec/cm^2^/sr), which are quantitative data obtained from a region of intensity (ROI) and include the photons emitted by bioluminescent cells from an assigned rectangular area over the whole body of each mouse.

### Bioinformatics

We used the Oncomine Cancer Microarray database (http://www.oncomine.org/) to analyze the expression levels of NCL in normal breast and colorectal tissues and in breast carcinoma and colorectal adenocarcinoma tissues. These gene expression data were log2 transformed and median centered. All graphics and statistical values were analyzed using GraphPad Prism 5.0, and *P*‑values were calculated by the two‑tailed Student's *t*‑test. Kaplan-Meier plots were generated using the R2 platform, and patients were grouped according to the NCL expression levels in their breast and colorectal tumors.

### Statistical analysis

All statistical data are expressed as the means ± SD (*n* = 3). Statistical comparisons between two groups were determined by Student's *t*-test, and comparisons among multiple groups were determined by one-way ANOVA with Dunnett's multiple comparison. For *in vivo* experiments, the number of mice is indicated in each legend. The log-rank test was used for Kaplan-Meier analyses. *, **, and *** indicate *P* < 0.05, *P* < 0.01, and *P* < 0.001, respectively.

## Results

### Identification and characterization of cancer-targeting peptide ligands by OBOC combinatorial screening and MAP synthesis methods

**Figure 1:**
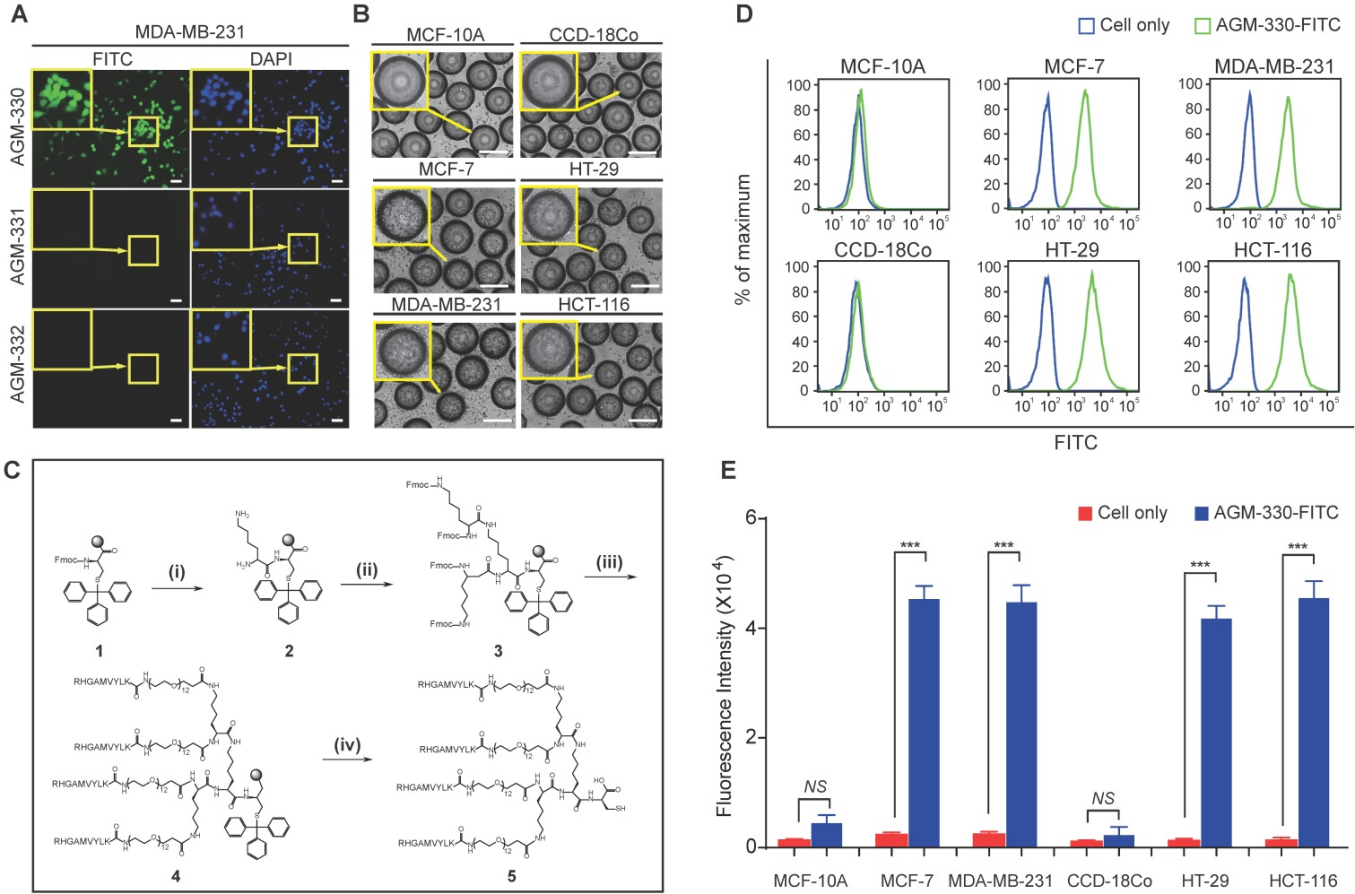
** Screening of OBOC libraries and MAP synthesis for cancer-specific ligands. A,** Binding specificity was determined by confocal imaging of the immunofluorescence of FITC-labeled AGM-330, AGM-331, and AGM-332. Nuclei were stained with DAPI (blue). Scale bar, 50 µm. **B,** Binding specificity of AGM-330. A whole-cell binding assay was performed to determine the cell binding of AGM-330 in serum free medium. Scale bar, 200 µm. **C,** Procedure for the synthesis of AGM-330. Note for reagents and conditions: (i) Fmoc-Lys-(Fmoc)-OH, piperidine, DMF; (ii) Fmoc-Lys-(Fmoc)-OH, piperidine, DMF; (iii) RHGAMVYLK-OH, piperidine; (iv) piperidine, DMF. Fmoc = 9-fluorenylmethoxycarbonyl, DMF = dimethylformamide. **D,** FACS analysis showing the specificity of AGM-330 toward cancer cells among multiple breast and colorectal cancer cells and normal breast and colorectal cells. **E,** Quantification of the FACS analysis results showing the specificity of AGHM-330. Bar graphs represent the mean ± SD, and statistical analyses were performed by one-way ANOVA with Dunnett's multiple comparison. *, **, and *** indicate *P* < 0.05, *P* < 0.01, and *P* < 0.001, respectively.

To identify a novel cancer-specific peptide ligand, we synthesized ~ 2,600,000 libraries using 5 g of Tentagel MB NH_2_ resin (200 μm, 520,000 beads/g; Figure S1A). A peptide library displayed on beads (50,000 ~ 100,000 beads were used at a time) was mixed with a human breast cancer cell line (MDA-MB-231). Of the ~ 1,000,000 beads that were screened, 8 positive beads were detected and isolated for microsequencing. Three of these 8 peptides (AGM-330, AGM-331, and AGM-332) showed strong preferential binding to MDA-MB-231 and showed no or only weak binding to the human normal breast cell line MCF-10A (Figure S1B-C). We then labeled these 3 peptides with fluorescein isothiocyanate (FITC) and determined whether they could bind to MDA-MB-231 cells. Compared with AGM-331 and AGM-332, the strongest fluorescence signals were detected with AGM-330 (Figure 1A). To confirm the screening results from the cell-growth-on-bead assay and fluorescence imaging, AGM-330 was resynthesized on Tentagel MB NH_2_ resin. A cell-growth-on-bead assay showed that the AGM-330 beads were fully covered by human breast cancer cell lines (MCF7 and MDA-MB-231) and human colorectal cancer cell lines (HT-29 and HCT-116) within 15 min (Figure 1B). In contrast, AGM-331 and AGM-332 were relatively nonspecific and bound to the human normal breast cell line MCF-10A and the human normal colorectal cell line CCD-18Co. In addition, AGM-330 bound either very weakly or not at all to MCF-10A or CCD-18Co cells, making them excellent candidates for both imaging and therapeutic targeting agents.

The use of peptides as therapeutic drugs has to date been largely limited by their low stability: peptides are primarily broken down by proteases and peptidases *in vivo* 21. In this study, to improve the stability of the peptide ligand, we synthesized AGM-330 in a MAP dendrimeric form, which can exhibit increased stability due to the acquired resistance to protease and peptidase activity. The synthesis of AGM-330 (Figure 1C) commenced with Fmoc-Cys(Trt) Wang resin (**1**) and Fmoc-Lys(Fmoc)-OH in the presence of 20% piperidine and DMF to provide lysine core-conjugated Wang resin (**2**,**3**). Then, Fmoc- and Trt-protected AGM-330 (**4**) was produced by treatment with RHGAMVYLK-PEG12-OH in the presence of 20% piperidine. The Fmoc and Trt protecting group in AGM-330 (**4**) was quickly removed by piperidine in DMF, giving rise to AGM-330 (**5**).

To evaluate the *in vitro* stability of peptides, monomeric AGM-330 (AGM-330m), dimeric AGM-330 (AGM-330d) and tetrameric AGM-330 (AGM-330) were incubated with serum, and the portion of peptide remaining was quantified by analytical HPLC. In consequence, more than 80% of the AGM-330 remained intact after 24 h incubation in serum, while, AGM-330m and AGM-330d were rapidly degraded by protease after incubation with serum (Figure S2A). These results showed that the tetrameric structure prevented enzymatic degradation 18-20. Accordingly, we defined tetrameric structure as the final potent cancer-specific peptide candidate. Next, we further examine the stability of AGM-330 *in vivo*. As a result, half-life of the tetrameric structure was 9.43 ± 1.21 h, which was remained more than 25% after 24 h treatment *in vivo* (Figures S2B-C).

Next, to examine whether the MAP ligand can selectively bind to cancer cells, cancer cells treated with FITC-labeled AGM-330 (AGM-330-FITC) were compared with untreated cells. AGM-330-FITC was incubated with the cells (1 × 10^5^) for 2 h at 37 °C in serum-free medium to keep the conjugates intact. Then, the inherent fluorescence of peptide-treated cells were compared and measured based on changes in the mean fluorescence intensity (MFI) values compared to those of untreated cells (Figure 1D and E). The results showed that AGM-330-FITC displayed significant cancer cell binding to MCF-7, MDA-MB-231, HT-29, and HCT-116, as evidenced by the increase in MFI of treated cells relative to the untreated cells. In contrast, the conjugates displayed a significant decrease in binding to normal cells, including MCF-10A and CCD-18Co, versus strong preferential binding to cancer cells after 2 h of incubation.

To examine whether AGM-330 was cytotoxic, we evaluated IC_50_ values in multiple cancer cells, including MCF-7, MDA-MB-231, HT-29, and HCT-116, and in normal cells, including MCF-10A and CCD-18Co. Cells were treated with different concentration of AGM-330 (0, 0.1, 1, 5, 10, 20, 50 and 100 μM) for 48 h. After 48 h of treatment with AGM-330, cell viability was not changed at concentrations up to 100 μM. The IC_50_ values of AGM-330 were greater than 100 μM in all cell lines (Figure S3A), demonstrating that AGM-330 does not appreciably affect the cell viability of cancer and normal cell lines. Furthermore, we determined whether serum affected the cancer cell binding and survival rate after AGM-330 treatment. The results showed that the presence (1 or 5% serum) or absence (0% serum) of serum did not affect cell survival or cancer cell specificity after AGM-330 treatment (Figure S3A-E). Taken together, these data indicate that AGM-330 can be utilized for the targeting of cancer cells without affecting target cell viability.

### NCL, a potent regulator of cancer cell growth, is a potential target of AGM-330

**Figure 2:**
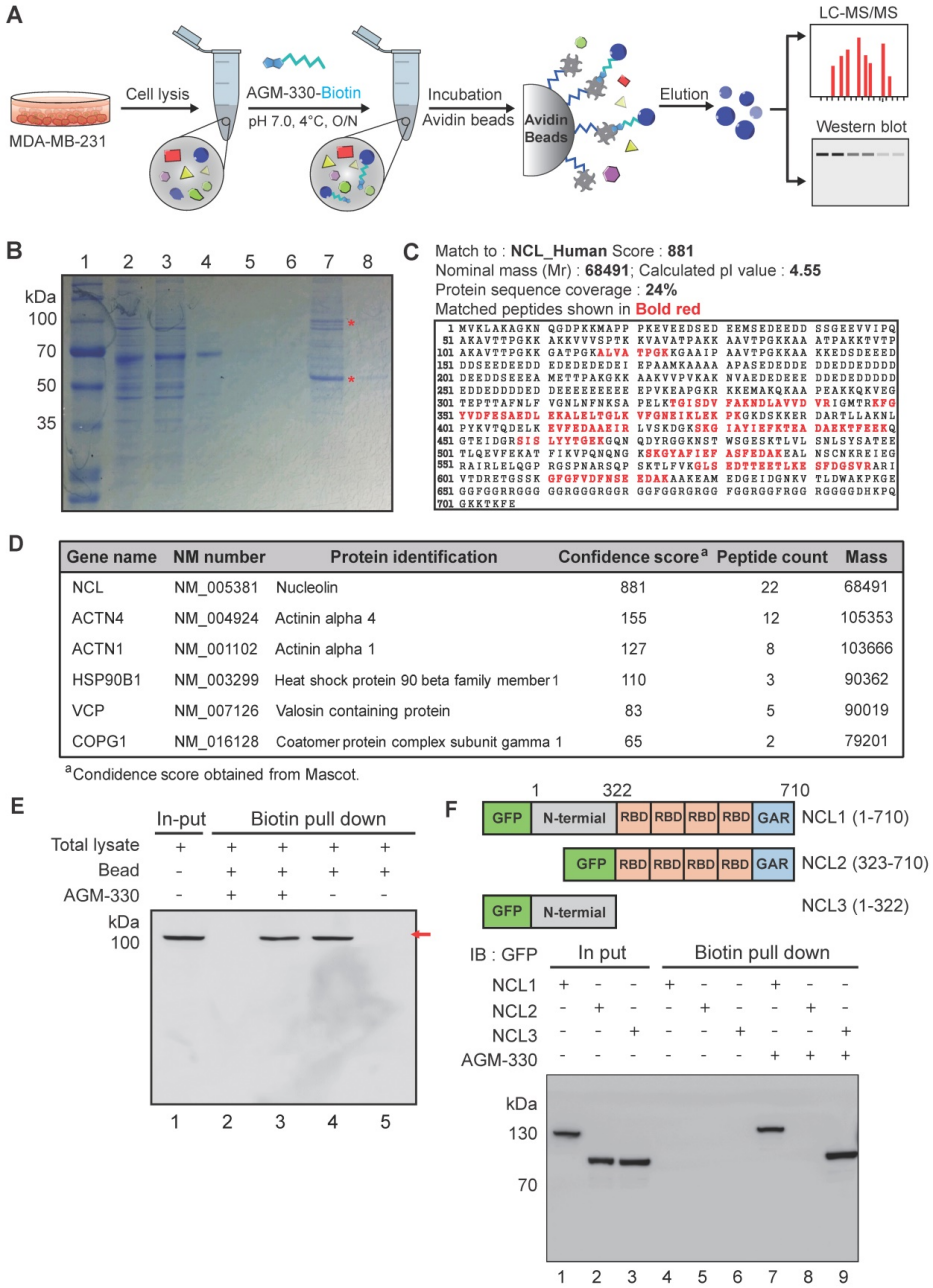
** Characterization and identification of molecular targets of AGM-330. A,** Schematic diagram of biotin pull-down assay, mass spectrometry analysis and Western blot. **B,** Coomassie brilliant blue staining of the eluted proteins from affinity columns after separation by 10% SDS-PAGE. Lane 1: protein marker; Lane 2: total lysate before biotin pull-down assay; Lane 3: flow-through after incubation with AGM-330-biotin; Lanes 4-6: bead washing fraction; Lanes 7-8: elution of AGM-330 binding proteins. * Red star indicates that the protein band was excised for in-gel digestion followed by LC-MS/MS analysis. **C,** Protein database searches of peptides detected by MS identified NCL as an AGM-330 binding partner. D, List of genes obtained from LC-MS/MS analysis. The confidence score indicates the mascot score of the identified protein. **E,** Immunoblotting analysis of the eluted proteins from the biotin pull-down assay using an anti-NCL antibody. The arrow indicates the presence of NCL. Lane 1: Input of total lysate; Lane 2: flow through after incubated with AGM-330-biotin; Lane 3: elution of AGM-330 binding proteins; Lane 4: flow through after incubated with beads (no AGM-330); Lane 5: elution of beads binding proteins (no AGM-330). **F,** Analysis of the NCL domains binding to AGM-330. Different GFP-NCL constructs were transfected into HEK293T cells. AGM-330-biotin was added to NCL residues 1-710, residues 1-322, or residues 323-710 bound to streptavidin beads. Proteins on the beads were analyzed using immunoblotting with anti-GFP antibodies. Lanes 1-3: Input of total lysate; Lanes 4-6: elution after incubation with beads (no AGM-330); Lanes 7-8: elution of AGM-330 binding proteins.

To identify the unknown target protein of AGM-330, affinity column chromatography and mass spectrometry-based proteomic approaches were employed to identify the AGM-330-interacting proteins from the cell lysate of MDA-MB-231 cells (Figure 2A). After affinity column chromatography, SDS-PAGE analysis revealed several distinct protein bands (from 55-100 kDa) that were only present in the elution fraction (Figure 2B). Six proteins were identified using LC-MS/MS analysis, and peptide identification of these excised bands showed that one of the major protein bands at 100 kDa was characterized as NCL. NCL comprises 710 amino acids, and 22 different peptide fragments that were identified by LC-MS/MS analysis were matched to the amino acid sequence of NCL (total score of 881 and 24% sequence coverage to human NCL in NCBI database (accession number: gi189306; Figure 2C and D). Moreover, immunoblot analysis using an anti-NCL antibody further confirmed the enrichment of NCL in the eluates from the AGM-330 affinity column (Figure 2E). Thus, the proteomic study results suggested that NCL is the AGM-330-interacting protein.

To further quantify the binding properties of AGM-330, surface plasmon resonance (SPR) analyses were performed with different concentrations of AGM-330. Under our experimental conditions, AGM-330 displayed a *K*_D_ value of 57.7 ± 12 nM (Figure S4). Next, to investigate the domain involved in the interaction between AGM-330 and NCL, we used the biotin pull-down assay. Biotinylated AGM-330 (AGM-330-biotin) was used as bait to pull-down NCL mutants. Several deletion mutants of NCL were generated. Cell extracts were prepared from HEK293T cells transfected with expression vectors encoding wild-type NCL and the various NCL mutants, including NCL1 (1-710), NCL2 (323-710) and NCL3 (1-322), all fused to GFP. AGM-330-biotin pulled down wild-type NCL1 (1-710) and NCL3 (1-322) but not NCL3 (323-710; Figure 2F). The results indicated that the N-terminal region (1-322) of NCL is important for its binding to AGM-330. Previous reports showed that NCL undergoes complex *N*-and *O*-glycosylations 22. In particular, NCL is glycosylated with two sialylated *O*-glycans among five potential *O*-glycosylation sites in TPXKK motifs of the N-terminal domain 23. To confirm whether the *O*-glycosylation status of the N-terminal domain of membrane NCL affects its interaction with AGM-330, the membrane fraction was separated and treated with *O*-glycosidase and α2-3,6,8,9 Neuraminidase A to obtain *O-*linked sialogycan-cleaved membrane NCL. As shown in Figure S5, a 100 kDa NCL band was shifted to 75 kDa after *O*-glycosidase and α2-3,6,8,9 neuraminidase A treatment. Next, to investigate the interaction between AGM-330 and sialogycan-cleaved membrane NCL, AGM-330-biotin was used as bait immobilized to streptavidin beads for pull-down assays. The immunoblot analysis results showed that NCL was enriched in the elution fractions. These data suggest that the AGM-330 does not interact with the glycan structures but selectively binds to the non-glycosylated structure of NCL (Figure S5). Taken together, these results indicate that AGM-330 directly interacts with NCL.

### AGM-330 specifically binds to cancer cells *in vitro* and *in vivo*

**Figure 3:**
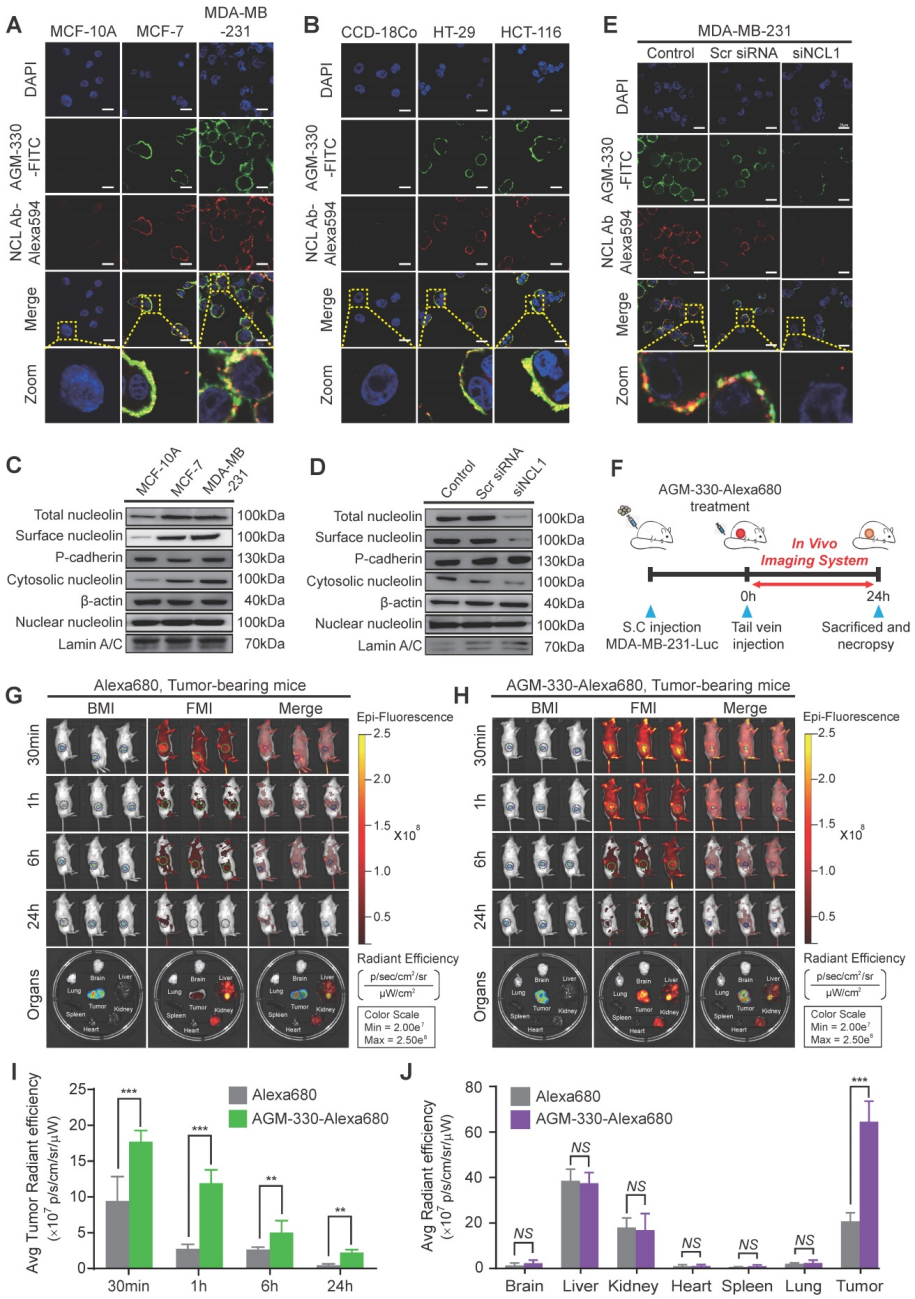
** Binding specificity of AGM-330 *in vitro* and *in vivo*. A, B;** Fluorescence confocal microscopy of multiple cancer and normal cells. MCF-10A, MCF-7, MDA-MB-231 (A) or CCD-18Co, HT-29, and HCT-116 (B) cells were incubated with 5 µmol/l AGM-330-FITC for 30 min at 37 °C. NCL was stained by primary anti-NCL antibody, and the complex was revealed using an anti-mouse secondary antibody coupled to Alexa Fluor 594. Cells were then fixed with 4% paraformaldehyde, and the nuclei were stained with DAPI. MERGE represents AGM-330-FITC, NCL, and nuclear staining by DAPI. AGM-330-FITC: FITC-conjugated AGM-330. Scale bar, 20 µm. **C,** The NCL distribution in various subcellular fractions was analyzed in MCF-10A, MCF-7, and MDA-MB-231 cells. Plasma membrane, cytosol and nuclear NCL were immunoblotted using anti-NCL antibody. **D,** NCL distribution in various subcellular fractions was analyzed in MCF-10A, MCF-7, and MDA-MB-231 cells after siNCL1 treatment. Plasma membrane, cytosol and nuclear NCL were immunoblotted using anti-NCL antibody. **E,** Binding of AGM-330 to the cell membrane is inhibited by knockdown of NCL using siRNA. MDA-MB-231 cells were transfected for 48 h. Then, 5 µmol/l of AGM-330-FITC was added for 2 h. Cells were washed twice with PBS and fixed with 4% paraformaldehyde. Fluorescence was observed using a fluorescent confocal microscope. AGM-330-FITC: FITC-conjugated AGM-330. Scale bar, 20 µm. **F,** Schematic representation of the experimental protocol as described in the materials and methods section. Anesthetized 6‑week-old male NPG^TM^ mice were inoculated with 1:1 mix of Matrigel and 1 × 10^6^ MDA-MB-231-luc cells into the mammary fat pad. After tumor cell inoculation, when the tumor volume reached approximately 100 mm^3^, free Alexa680 or AGM-330-Alexa680 was injected into the tail vein. **G, H;** *In vivo* fluorescence images of mice 30 min, 1 h, 6 h, and 24 h after intravenous injections of 10 nmol free Alexa680 (G) or 10 nmol AGM-330-Alexa680 (H) in MDA-MB-231-luc tumor-bearing mice. Fluorescence images of the major organs including the heart, spleen, lung, brain, liver, kidney, and tumors removed from mice with different treatments. AGM-330-FITC: FITC-conjugated AGM-330. **I, J;** Average radiant efficiency of Alexa680-associated fluorescence from live imaging of three animals in tumors (I) and different organs (J). Bar graphs represent the mean ± SD, and statistical analyses were performed by one-way ANOVA with Dunnett's multiple comparison. *, **, and *** indicate *P* < 0.05, *P* < 0.01, and *P* < 0.001, respectively.

We evaluated the specificity of AGM-330 to NCL with cell binding assays against different cell lines using fluorescence microscopy. The experiment was performed using multiple cancer cell lines, MCF-7, MDA-MB-231, HT-29, and HCT-116, and normal cell lines, MCF-10A, and CCD-18Co. The binding specificity was determined by confocal imaging after 1 h of incubation of the cells with 5 μmol/l AGM-330-FITC. The cells were incubated with DAPI to counterstain the nuclei (blue fluorescence). Double-immunofluorescence staining with an anti-NCL antibody that binds to the N-terminal domain of NCL and AGM-330-FITC revealed an overlap between NCL and AGM-330-FITC, indicating that AGM-330 binds to NCL (Figure 3A and B). A previous study showed that the pseudopeptide N6L bound to membrane NCL and became internalized into the nucleolus of cancer cells 24. To evaluate the ability of AGM-330 to undergo NCL-mediated endocytosis, MDA-MB-231 cells were incubated with AGM-330-FITC at 37 °C for different times (1, 3, 6 and 12 h). The results confirmed that AGM-330 was primarily distributed on the cell membrane and was not taken up by the cytoplasm or nucleolus (Figure S6). As mentioned in a previous study, N6L is essentially built on a lysine-rich helicoid template formed by a repeat of 6 tripeptide units. However, as shown in Figure 1C, AGM-330 showed tetra-branched structures conjugated by a trilysine core. Therefore, the difference in the molecular structure between AGM-330 and N6L may be caused by different endocytosis mechanisms. In addition, previous data indicated that N6L is able to interact with both NCL and nucleophosmin, where knockdown of nucleophosmin by siRNA was shown to decrease N6L translocation from the membrane to the nucleolus. This result provides strong evidence that nucleophosmin is involved in N6L internalization. However, except for membrane NCL, we could not confirm the protein associated with internalization among the proteins bound to AGM-330 (Figure 2D). Taken together, N6L, which binds to both membrane NCL and nucleophosmin, induce apoptosis after internalization into nucleolus of cancer cells. However, AGM-330, which binds to only membrane NCL, was not internalized into cytoplasm or nucleolus and did not affect cell viability (Figure S3A).

Next, we determined the relative amounts and intracellular localization of NCL in MCF-7, MDA-MB-231, and MCF-10A cells to determine whether differences in either the expression level or localization of NCL were related to the sensitivity of cancer cells to AGM-330. We separated the subcellular fraction of cells and analyzed the expression of NCL in the cytosolic, nuclear, and cell membrane extracts, as well as the total NCL in cells, by Western blotting. The results of this analysis revealed that NCL expression was elevated in membrane and cytoplasmic extracts of MCF-7, MDA-MB-231, HT-29, and HCT-116 cells compared with that observed in MCF-10A and CCD-18Co cells (Figures 3C and S7A). No significant differences in the nuclear levels of NCL were observed among all cell lines.

To investigate whether membrane-expressed NCL is critical for AGM-330 binding to cancer cells, we used specifically targeting siRNAs for NCL knockdown. We observed that three siRNAs targeting NCL (siNCL1, siNCL2, and siNCL3) showed different efficacies in NCL-knockdown cells. Because siNCL1 showed the highest knockdown efficacy in MDA-MB-231 cells, it was selected for further use (Figure S7B). Next, to confirm whether NCL expression at the cancer cell membrane is decreased after siNCL1 treatment, we separated the subcellular fraction of siNCL1-treated cells and analyzed the expression of NCL in the cytosolic, nuclear, and cell membrane extracts by Western blotting. The results showed that siNCL1 treatment potently inhibited the basal expression of NCL in cytosolic and cell membrane fractions (Figure 3D). Following expression level studies of NCL, we confirmed that decreased expression of membrane NCL affected AGM-330 binding. As a result, scrambled siRNA-treated cells showed similar fluorescence signals as controls. However, little or no fluorescence signal was detected in the siNCL1-treated group due to the decreased AGM-330 binding (Figure 3E). Fluorescence imaging (Figure 3A and B) results revealed that the anti-NCL antibody, which binds to the membrane of cancer cells, and AGM-330-FITC colocalized with anti-NCL antibody fluorescence. Additionally, mutant analysis showed that the N-terminal domain of NCL was responsible for the interaction between NCL and AGM-330 (Figure 2F). Accumulating evidence suggests that AGM-330 directly interacts with NCL, which is expressed in the membranes of cancer cells.

Next, we used a xenograft model of human breast cancer to evaluate the cancer-targeting properties of AGM-330 by *in vivo* imaging (Figure 3F). To examine whether AGM-330 can selectively target cancer cells *in vivo*, tumor-bearing mice treated with Alexa680 labeled AGM-330 (AGM-330-Alexa680) were compared to mice treated with Aleax680 alone. The near-infrared fluorescent (NIRF) intensities of tumors 1 h after AGM-330-Alexa680 treatment were significantly higher than those observed for control tumors: 3.04 × 10^7^ ± 1.38 10^6^ versus 1.19 × 10^9^ ± 1.90 10^7^ (p/sec/cm^2^/sr)/(μW/cm^2^) for the control and AGM-330-Alexa680-treated tumors, respectively; *n* = 3, *P* < 0.001 (Figure 3G-I). The percentage of the injected dose of AGM-330-Alexa680 and of Alexa680 alone in the tumor and other major viscera at 2 h was also analyzed to quantitatively examine the effect of AGM-330 on the distribution of the conjugated fluorescent. Interestingly, the NIRF total photon counts per gram of each organ from the tumor tissues, except the kidney and liver, were significantly higher than those observed for other organs, providing definitive evidence that the tumor-targeting activity of AGM-330 is much greater than that of the controls (Figure 3J). Consistent with a previous report 25, the *ex vivo* results showed that Alexa680 was primarily distributed in the livers and kidneys of non-tumor bearing mice. The fluorescence intensity was the strongest at 30 min post-injection and then gradually decreased by clearance 24 h after injection (Figure S7C). Taken together, the NIRF images revealed that AGM-330 primarily accumulated throughout the tumor tissue rather than normal organs *in vivo*.

### NCL is positively correlated with cancer progression, and anti-NCL antibodies effectively inhibit cell proliferation via membrane NCL neutralization

**Figure 4:**
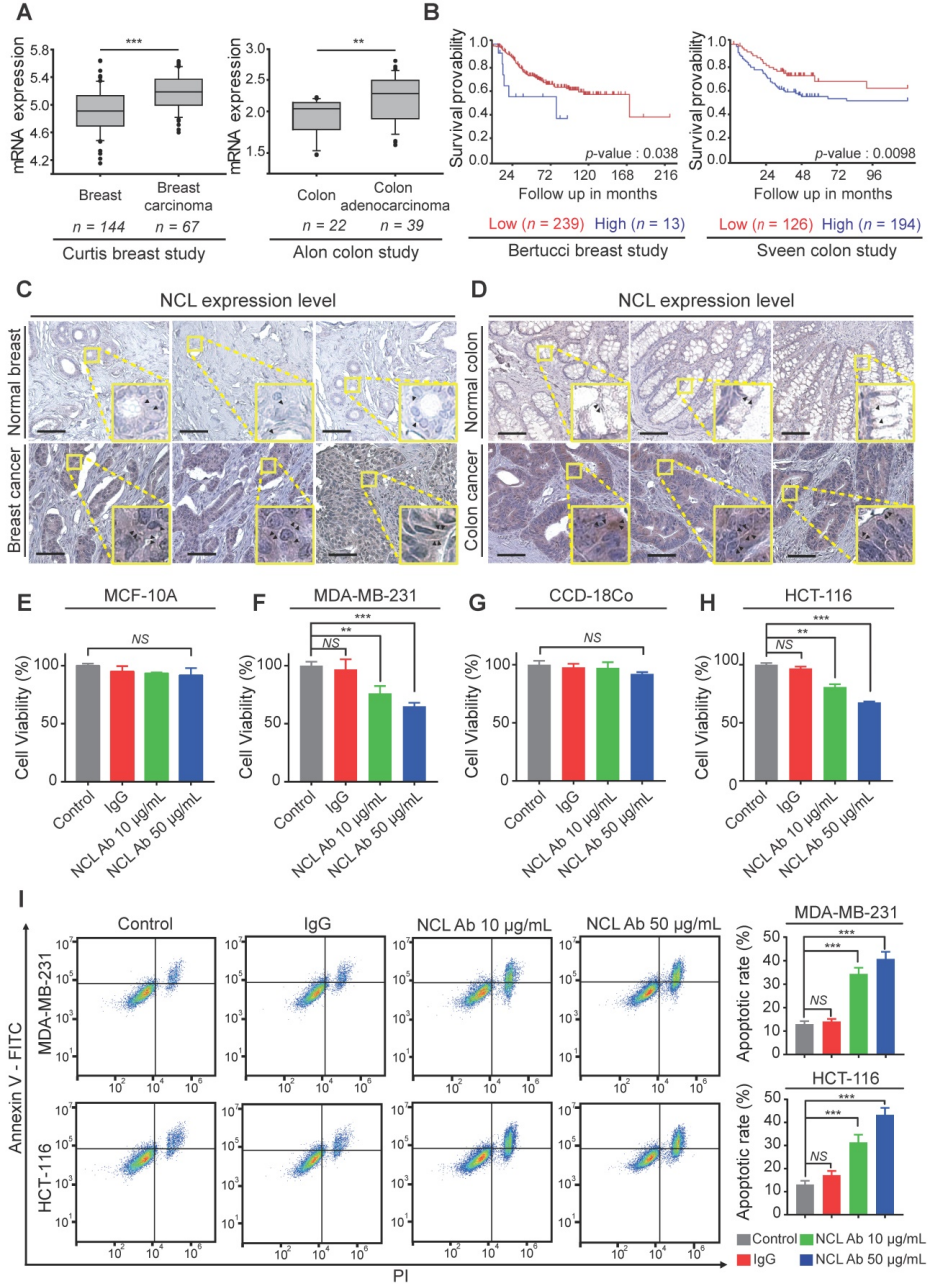
** Expression profiling and functional study of NCL in breast and colorectal cancer. A,** A significant correlation between tumor occurrence breast carcinoma or colorectal adenocarcinoma patients and the expression levels of NCL mRNA was observed in the Curtis and Alon datasets, which were obtained through the Oncomine dataset repository (www.oncomine.org). **B,** Kaplan-Meier survival analyses were conducted for patients with breast and colorectal cancer based on NCL expression in two independent cohorts (Bertucci and Sveen). **C, D,** Immunohistochemistry assay (IHC) for NCL in normal breast and breast cancer tissues (C) and normal colon and colon cancer tissues (D). Scale bar, 100 µm. **E-H,** Cancer and normal cells were treated with the indicated amounts of anti-NCL antibody or IgG in serum-free media for 24 h. Cell viability of MCF-10A (E), MDA-MB-231 (F), CCD-18Co (G), and HCT-116 (H) was measured by WST assay. I, Apoptotic cells were visualized by staining Annexin V^+^ cells. Cells were neutralized with the anti-NCL antibody and incubated in serum-free media for 24 h. Apoptotic cells were measured by FACS analysis. Bar graphs represent the mean ± SD, and statistical analyses were performed by one-way ANOVA with Dunnett's multiple comparison. *, **, and *** indicate *P* < 0.05, *P* < 0.01, and *P* < 0.001, respectively.

Based on the above results, we confirmed the NCL expression pattern in cancer cell lines. Next, to examine NCL expression in human cancer tissues, we analyzed the available breast and colorectal cancer datasets using the Oncomine dataset repository (www.oncomine.org). The relative expression of NCL in 144 primary breast tissues versus 67 breast carcinomas was analyzed in the Curtis datasets. NCL mRNA expression was significantly upregulated in breast carcinoma compared to normal breast tissue (*P* < 0.001, Figure 4A). Additionally, the expression of NCL mRNA was significantly (*P* < 0.01, Figure 4A) higher in colorectal adenocarcinoma than in the normal counterpart tissue in the Alon datasets. Next, we observed that high NCL expression was associated with poor prognosis, such as low disease-free survival, in patients with breast and colorectal cancer (Bertucci and Sveen dataset from 'R2: Genomics Analysis and Visualization platform (http://r2.amc.nl)'; Figure 4B), which is consistent with a previous report showing that high NCL expression serves as a poor prognostic marker in patients with breast and colorectal cancer 26,27. To determine the distribution of NCL expression in human cancer and normal tissues, we analyzed NCL expression by immunohistochemistry (IHC) analysis. Consistent with the results of immunofluorescence imaging (Figure 3A and B) and Western blotting (Figure 3C), IHC results revealed NCL levels were also increased in the membrane and cytoplasm regions of human breast and colorectal cancer tissues compared with those observed in normal breast and colorectal tissues (Figure 4C and D). In addition, previous reports have shown that upregulated membrane NCL interacts as a receptor for ligands involved in cancer proliferation and the inhibition of apoptosis 28,29. Therefore, to examine whether membrane-expressed NCL is essential for regulating functions in cancer cells, we neutralized membrane-expressed NCL using monoclonal anti-NCL antibodies on MDA-MB-231 and HCT-116 cells and MCF-10A and CCD-18Co cells *in vitro*. First, cell viability was evaluated on normal and cancer cells treated with antibodies at different concentrations for 24 h. Treatment with the NCL antibody (50 μg/ml) led to 35 and 32% reductions in cell viability in MDA-MB-231 and HCT-116 cells, respectively, but no significant decrease was observed in normal cells compared to the control and IgG treated groups (MCF-10a and CCD-18Co; Figure 4E-H). Additionally, we performed apoptosis assays to examine the cause of the cytotoxicity effect of the anti-NCL antibody on cancer cells. Annexin V and PI were used to stain MDA-MB-231 and HCT-116 cells treated with antibodies at different concentrations at 24 h. Surprisingly, the flow cytometry results revealed that the percentages of cancer cells in the early and late apoptosis phases increased in an anti-NCL antibody dose-dependent manner (Figure 4I). Interestingly, although treatment with anti-NCL antibodies induced apoptosis in cancer cells, treatment with AGM-330 produced no change in cancer cell viability (Figure S3A-C). A previous study showed that application of anti-NCL antibodies, but not control IgG, abrogated the PI3K/Akt phosphorylation in cancer cells 28. These results revealed that membrane NCL is involved in the PI3K/Akt signaling in cancer cells. To examine the difference in toxicity between the anti-NCL antibody and AGM-330, we examined the effect of the anti-NCL antibody and AGM-330 on PI3K/Akt signaling by immunoblot analysis. As a result, treatment of anti-NCL antibody suppressed PI3K/Akt phosphorylation in concentration dependent manner (Figure S8A-B). In contrast, AGM-330 did not suppress PI3K/Akt phosphorylation (Figure S8C-D). Taken together, after binding to membrane NCL, anti-NCL antibodies suppressed PI3K/Akt phosphorylation and induced cancer cell apoptosis. However, AGM-330 did not suppress PI3K/Akt phosphorylation, suggesting that AGM-330 just binds to the membrane NCL without altering cell viability.

In this study, we confirmed the overexpression of both membrane and cytoplasmic NCL in various types of cancer cells compared to normal cells. Moreover, similar to previous studies which suggested the significant correlation between increased NCL expression and cancer aggressiveness, our finding supports the correlation of NCL overexpression with poor prognosis survival in cancer patient. Taken together, these results indicate that targeting NCL is a promising and efficient approach for cancer therapy. Therefore, we suggest that AGM-330 peptide is novel molecule that targets NCL with promising potential for use as a tool in cancer theranostics.

### Paclitaxel-conjugated AGM-330 effectively inhibits cancer growth *in vitro* and in a mouse xenograft model

**Figure 5:**
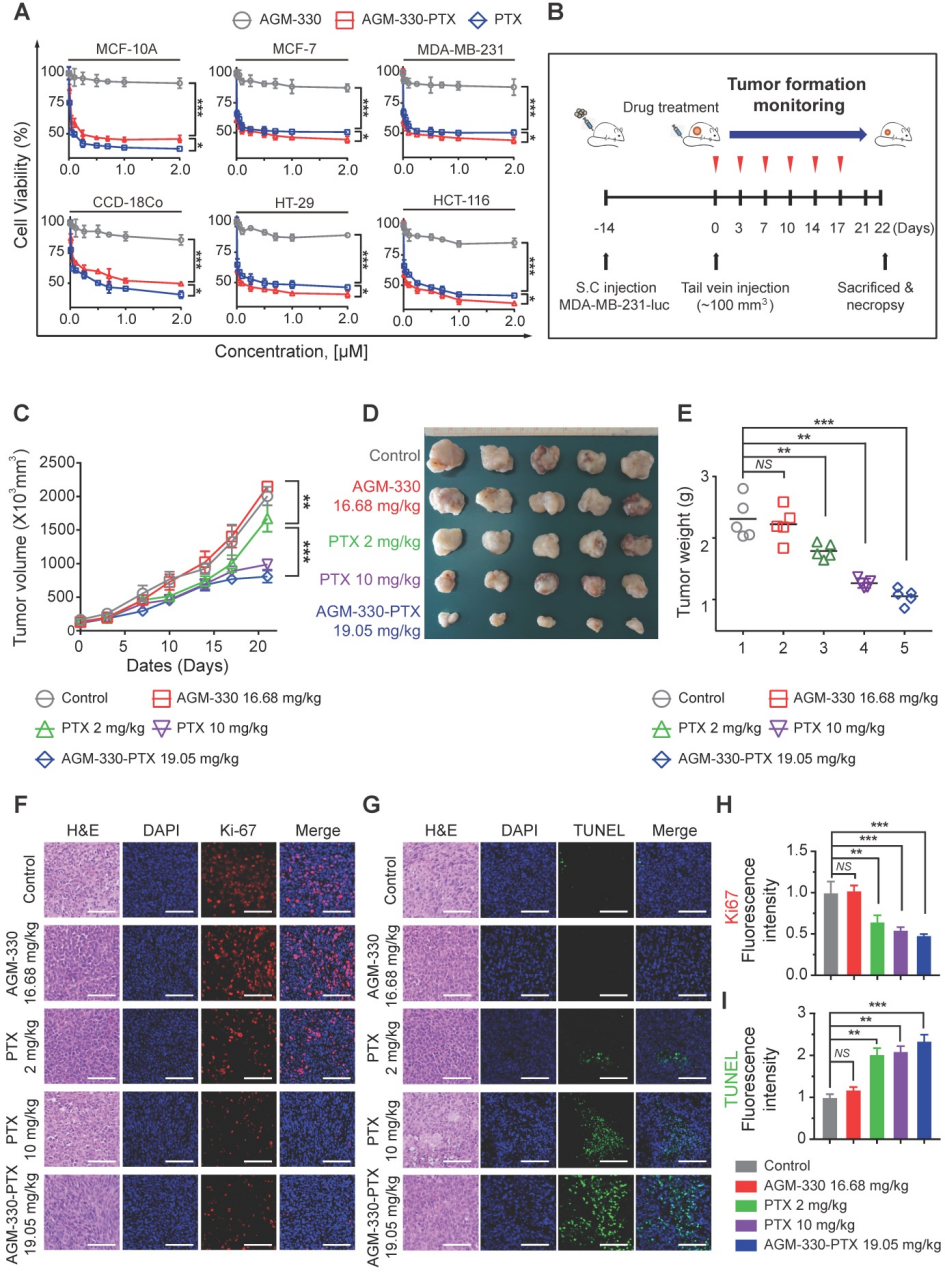
** AGM-330 led to increased therapeutic efficacy of PTX *in vivo*. A,** Cell proliferation inhibition by AGM-330-PTX treatment was determined for 48 h by the WST assay in multiple breast and colorectal cancer cells and in normal cells. **B,** Schematic representation of the experimental protocol as described in the materials and methods section. Anesthetized 6‑week-old male NPG mice were inoculated with a 1:1 mix of Matrigel and 1 × 10^6^ MDA-MB-231-luc cells into the mammary fat pad. When the volume of primary tumors reached approximately 100 mm^3^, tumor-bearing mice were treated with vehicle (PBS), PTX (2 or 10 mg/kg), AGM-330 (16.68 mg/kg, molar equivalent to 2 mg/kg of PTX), or AGM-330-PTX (19.05 mg/kg, molar equivalent to 2 mg/kg of PTX; *n* = 5/group). **C-E,** The therapeutic effect of AGM-330-PTX was evaluated in breast cancer xenograft models. C, Primary tumor volume was measured twice a week until the day of sacrifice. The primary tumor volume was calculated by the formula volume (mm^3^) = (length (mm)) × (width (mm))^2^ × 0.5. D, On the day of sacrifice, all primary tumors were isolated, and (E) the primary tumor weights were evaluated. **F-I,** Immunohistochemistry (IHC) sections of representative tumors with Ki-67 (F,H) and TUNEL (G,I) staining. Nuclei were stained with DAPI (blue). Scale bar, 50 µm. Bar graphs represent the mean ± SD, and statistical analyses were performed by one-way ANOVA with Dunnett's multiple comparison. *, **, and *** indicate *P* < 0.05, *P* < 0.01, and *P* < 0.001, respectively.

Paclitaxel (PTX) was conjugated with AGM-330 (AGM-330-PTX) to obtain a cancer-targeting ligand linked to a well-established chemotherapeutic agent. The chemical reaction between the maleimide groups present in the PTX and the thiol group in AGM-330 was optimal at a maleimide to thiol molar ratio of 10:1. In addition, AGM-330 and maleimide-PTX combined at a ratio of 1:1. Maleimide-PTX, AGM-330 and AGM-330-PTX were characterized by HPLC and MS (Figure S9A-J). In this study, a propionic acid linker was incorporated between PTX and AGM-330 to facilitate the release of PTX through a spontaneous hydrolysis reaction. These linkers have been reported to be rapidly hydrolyzed in serum 30. To examine the release of PTX after linker hydrolysis, AGM-330-PTX was incubated in serum for different times (5, 30 min, 1, 2, 6 and 24 h). The presence of intact AGM-330-PTX and released moieties (AGM-330) after hydrolysis were assessed by HPLC. AGM-330-PTX was almost completely hydrolyzed after 2 h (Figure S10A). We additionally confirmed that the AGM-330-PTX was fully hydrolyzed in PBS after 12 h, indicating that enzymes contained in serum promote the hydrolysis of the linker (Figure S10B). Molecular mass analysis was performed using MALDI-TOF MS (Shimadzu; Figure S10C-D). To determine whether the released moieties were taken up by cancer cells, rhodamine B-labeled PTX (PTX-Rh) and PTX-Rh-conjugated AGM-330 (AGM-330-PTX-Rh) were synthesized and characterized by NMR, HPLC and MS (Figure S9K-N). In addition, to confirm whether AGM-330 was internalized into cancer cells, AGM-330-specific antibodies were isolated from the immunized rabbits (Young in Frontier). Subsequently, MDA-MB-231 cells were incubated with AGM-330-PTX-Rh for different times and analyzed by confocal microscopy. After 30 min, double-immunofluorescence staining with an anti-AGM-330 antibody that binds to AGM-330 and Rh revealed an overlap between AGM-330 and PTX-Rh, indicating that intact AGM-330-PTX-Rh binds to membrane NCL (Figure S10E). However, after a 2 h incubation, the confocal microscopy results showed that AGM-330 was prominently localized on the cell membrane, and the hydrolyzed PTX-Rh moieties were internalized into MDA-MB-231 cells (Figure S10E). Taken together, these results confirmed that AGM-330 was primarily localized on the cell membrane and that PTX was internalized into cancer cells after the hydrolysis of the propionic acid linker, suggesting that NCL-specific binding of AGM-330 enables its release to allow for the entry of PTX into cancer cells. Next, to examine whether AGM-330-PTX inhibits cancer cell proliferation, we evaluated its IC_50_ values in multiple cancer cell lines, including MCF-7, MDA-MB-231, HT-29, and HCT-116 cells (Figure 5A). In MDA-MB-231 cells, the IC_50_ values for PTX alone and AGM-330-PTX were 0.37 and 0.26 μM, respectively. The results were similar in all three cancer cell lines (MCF-7, HCT-116, and HT-29). However, in MCF-10A cells, the IC_50_ values for PTX alone and AGM-330-PTX were 0.45 and 0.77 μM, respectively.

Following *in vitro* proliferation analysis on cancer lines, we investigated whether AGM-330-PTX could be a potential therapeutic agent *in vivo*. We inoculated MDA-MB-231-luc cells into the mammary fat pad of NPG mice. After the subcutaneous tumors grew to 100 mm^3^, comparative efficacy studies were performed by dividing the animals into five groups (*n* = 5/group) treated with vehicle (PBS), PTX (2 or 10 mg/kg, respectively), AGM-330 (16.68 mg/kg, molar equivalent to 2 mg/kg of PTX), or AGM-330-PTX (19.05 mg/kg, molar equivalent to 2 mg/kg of PTX) twice a week by tail vein injection (Figure 5B). Tumor growth was monitored by measuring volumes twice a week with a caliber rule over the course of 3 weeks. Consistent with *in vitro* experiments, vehicle (PBS)-treated mice and AGM-330-treated mice showed no significant difference in the average tumor volume. However, the average tumor volume of mice treated with AGM-330-PTX (807 ± 90 mm^3^ for 19.05 mg/kg, *n* = 5) had decreased by approximately 52% with respect to animals treated with the same dose PTX (1671 ± 199 mm^3^ for 2 mg/kg, *n* = 5, *P* < 0.001). Furthermore, AGM-330-PTX-treated mice showed enhanced drug efficacy compared to the 5-fold increased dose of PTX-treated mice at day 21 (988 ± 78 mm^3^ for 10 mg/kg, *n* = 5, *P* < 0.05; Figure 5C and D). Consistent with the above results, the final average tumor weight in mice treated with AGM-330-PTX (1.3 ± 0.1 g for 19.05 mg/kg,* n* = 5) was significantly decreased compared to mice injected with PTX (2.1 ± 0.4 and 1.5 ± 0.1 g for the 2 and 10 mg/kg treatment groups, respectively; *n* = 5; Figure 5E).

We also performed histological staining of the excised tumors, and an independent pathologist evaluated the slides. Median tumors excised from AGM-330-PTX-treated mice exhibited a reduction in the number of cancerous cells and an increase in apoptotic cell nuclei, as demonstrated by H&E staining, Ki-67 and TUNEL assays, respectively (Figure 5F-I). In contrast, the vehicle (PBS)- and AGM-330-treated tumors contained larger numbers of tumor cells and exhibited few signs of apoptosis. Furthermore, mouse body weights were not affected by AGM-330-PTX administration compared to the mice administered vehicle (PBS), and the blood chemistry analysis results revealed no adverse signs of toxicity in the AGM-330-PTX-treated mice (Figure S11A-F). Accordingly, these findings suggest that the tumor specificity of AGM-330 may enhance the efficacy of PTX without detectable side effects resulting from off-target binding. In summary, membrane NCL-targeted AGM-330 may have potential as a molecular tool in the diagnosis and treatment of cancer.

## Discussion

The discovery of peptide ligands that can functionally and specifically target tumors offers a new opportunity in cancer diagnosis and treatment 31. However, the use of peptide ligands has largely been limited by their short biological half-life. In this study, using OBOC screening and MAP synthesis methods, we discovered a novel cancer-specific peptide ligand, AGM-330, and we observed that the synthesis of AGM-330 dramatically increased the stability of the ligand *in vivo* (Figure S2B). Additionally, we showed that the cancer specificity of AGM-330 may enhance the efficacy of PTX, increasing the therapeutic PTX concentration in tumor tissues (Figure 5C-E). Furthermore, we successfully identified NCL as the target protein of AGM-330 by pull-down assays and LC-MS/MS (Figure 2E). Thus, the current study suggests OBOC combinatorial library screening and the MAP synthesis method as a potential strategy for cancer-specific peptide discovery and AGM-330 as a lead compound for developing novel peptide carriers in cancer therapy.

In this study, AGM-330 specifically bound to cancer cells but bound weakly or not at all to normal breast and colorectal cells. These results strongly suggest that AGM-330 is cancer-specific, making it a prime candidate for the development of cancer-targeting peptides. In particular, in a mouse xenograft study, AGM-330 treatment showed more accumulation in tumors than in the heart, spleen, lung or brain. The specific targeting ability of AGM-330 to cancer cells provides a potential molecular tool for the diagnosis of cancer. In a human breast cancer xenograft mouse model, AGM-330-PTX treatment resulted in 63 and 43% decreases in the volume and weight, respectively, of tumors compared with those observed in the PBS-treated group (Figure 5C-E). The efficient targeting ability of AGM-330 to cancer cells and tissues provides a promising way to deliver anti-cancer drugs for cancer treatment. We believe AGM-330 is an ideal ligand for the delivery of anticancer drugs to cancer cells and therefore has great potential to become a new, effective therapeutic tool for cancer treatment. Furthermore, if the effect of AGM-330-PTX was further validated in the patient-derived tumor (PDT) model, this validation could be used to guide future use of AGM-330-PTX in clinical use with potential approach as anti-cancer drug.

PTX is considered one of the most promising cancer chemotherapy drugs and has been tested against many different human malignancies 32-34. However, treatment with PTX has certain limitations. Clinical development has been impeded by the high hydrophobicity of PTX and related difficulties with its formulation. Many side effects associated with PTX treatment have also been ascribed to the dilution solvents used in the formulation 35-37. However, unlike PTX, AGM-330 conjugated PTX is highly hydrophilic. This hydrophilicity may improve its pharmacokinetic profile and enable the use of smaller amounts of toxic dilution buffers, thus further decreasing the general toxicity of chemotherapy. Our results suggest that AGM-330-mediated targeted delivery may not only be less toxic but also more effective.

Although NCL is predominantly localized in the nucleus of normal cells, previous studies showed enhanced overexpression of membrane NCL in many different types of cancer cells 38-45. For instance, a two-fold increase in NCL expression is observed in tumor tissue from colorectal cancer human patients 46. In tumor tissue from breast cancer, NCL is also overexpressed, and high amounts of NCL are associated with a worse prognosis for patients 47. Consistent with these reports, we analyzed the available cancer data repositories in the Oncomine database (www.oncomine.org) and validated a significant correlation between the expression of NCL and the progression of breast and colon cancers (Figure 4A). Furthermore, we confirmed the NCL expression by IHC analysis in patient-derived tumor tissues. The IHC results showed that membrane NCL was significantly increased in human breast and colon cancer tissues compared with that observed in normal breast and colon tissues (Figure 4C and D). Because of its selective expression in various cancers and its oncogenic relevance, NCL has been suggested as a novel target for anti-cancer treatment. Accordingly, the use of molecules targeting NCL may be an effective approach for the selective delivery of drugs to tumors while minimizing side effects 48-50.

Several groups have developed molecules, such as aptamers (AS1411), pseudopeptides (HB-19 and N6L) or antibodies (4LB5) that bind to membrane NCL in cancer cells 4,24,40,48. These compounds have also been suggested as potential carriers for the targeted delivery of several anti-cancer drugs to cancer cells. However, although promising, aptamers, pseudopeptides and antibodies suffer from intrinsic limitations, such as extremely short half-lives, undesired immunostimulatory activities, and unknown toxicological effects. In contrast, peptide ligands have many advantages, such as the capability for large scale synthesis, low immunogenicity, the generation of non-toxic metabolites, and high *in vivo* biocompatibility. In general, peptide was not stable and degraded in a few minutes due to presence of endopeptidase and endoprotease *in vivo* 51. However, we confirmed that *in vivo* half-life of the AGM-330 was 9.43 ± 1.21 h (Figures S2B-C). Furthermore, we showed that the tumor specificity of AGM-330 enhanced the delivery efficacy of the anti-cancer drug *in vitro* and *in vivo*. Taken together, these results suggest that AGM-330 is a novel peptide-based NCL-targeting molecule with great potential for use as a tool in cancer treatment.

Conjugation with polyethylene-glycol (PEG), known as PEGylation, has been widely used to improve the pharmacological properties of therapeutic proteins 52-54. In this study, we used PEGylation in the synthesis of AGM-330 to decrease steric hindrance and increase hydrophilicity. However, PEG has been burdened by immunogenicity, which has a negative clinical effect on therapeutic molecules 55-57. A previous study suggested that the anti-PEG immune response to the PEGylated molecules depended on the immunogenicity of the extent of PEGylation and the molecular weight of PEG 58. To reduce the effects of PEGylation immunogenicity, we tested the anticancer efficacy using AGM-330d-PTX, which had a lower extent of PEGylation than AGM-330 (Figure S12A). In this study, AGM-330d-PTX-treated mice showed a dramatic reduction in luciferase activity compared with that observed in PTX-treated mice (Figure S12C-D). Additionally, AGM-330d-PTX treatment resulted in a significant decrease in the volume and weight of tumors compared with that observed in the PTX-treated group (Figure S12E-G). Consistent with the results obtained using AGM-330-PTX, mouse body weights were not affected by AGM-330d-PTX administration compared to that observed in the vehicle (PBS)-treated mice (Figure S12H). Although reduced stability was observed (Figure S2), the cancer specificity of AGM-330d might have enhanced the efficacy of PTX, increasing the therapeutic drug concentration in tumor tissues. Further PEG optimization is required to identify the best candidates for *in vivo* applications.

## Conclusions

In summary, by screening for cancer specific peptide ligands, we identified AGM-330 as a novel NCL-targeting peptide. In particular, we showed that AGM-330-PTX has favorable properties and possesses powerful antitumor activity *in vivo*. Taken together, our results show the great potential of AGM-330 as prospective pharmacological tool in cancer treatment.

## Supplementary Material

Supplementary figures and tables.

## Abbreviations

BSA: Bovine serum albumin
DIC: N,N'-Diisopropylcarbodiimide
FACS: Fluorescence-activated cell-sorting
FBS: Fetal bovine serum
FITC: Fluorescein isothiocyanate
Fmoc: 9-Fluorenylmethoxycarbonyl
HOBt: N-Hydroxybenzotriazole
IACUC: Institutional Animal Care and Use Committee
IHC: Immunohistochemistry
IRB: Institutional Review Board
MALDI-TOF-MS: Matrix-assisted laser desorption ionization time-of-flight mass spectrometry
MAP: Multiple-antigen-peptide
MFI: Mean fluorescence intensity
NCL: Nucleolin
NIRF: Near-infrared fluorescent
OBOC: One-bead-one-compound
PEG: Polyethylene-glycol
PI: Propidium iodide
PTX: Paclitaxel
ROI: Region of intensity
