# Supplementary material for: A novel nucleolin-binding peptide for Cancer Theranostics
Source: Theranostics. 2020 Jul 14;10(20):9153–71. doi: 10.7150/thno.43502 (PMC7415810; doi:10.7150/thno.43502)
Supplement: Supplementary file 1 — Supplementary figures and tables. [file thnov10p9153s1.pdf]

Supplementary Materials

A novel nucleolin-binding peptide for cancer theranostics

Jae-Hyun Kim[1], Chanhyung Bae[2], Min-Jung Kim[1], In-Hye Song[1], Jae-Ha Ryu[3], Jang-Hyun Choi[1], Choong-Jae Lee[1], Jeong-Seok Nam[1,*] and Jae Il Kim[1,3,*]

[1]School of Life Sciences, Gwangju Institute of Science and Technology, Gwangju 61005, Republic of Korea

[2]Molecular Physiology and Biophysics Section, Porter Neuroscience Research Center, National Institute of Neurological Disorders and Stroke, National Institutes of Health, Bethesda, United States

[3]Pilot Plant, Anygen, Gwangju, Technopark, 333 Cheomdankwagi-ro, Buk-gu, Gwangju, 61008, Republic of Korea

*Corresponding authors

jikim@gist.ac.kr (e-mail), +82-62-715-2494 (phone), +82-62-715-2484 (fax)

namje@gist.ac.kr (e-mail), +82-62-715-2893 (phone), +82-62-715-2484 (fax)

Figure S1

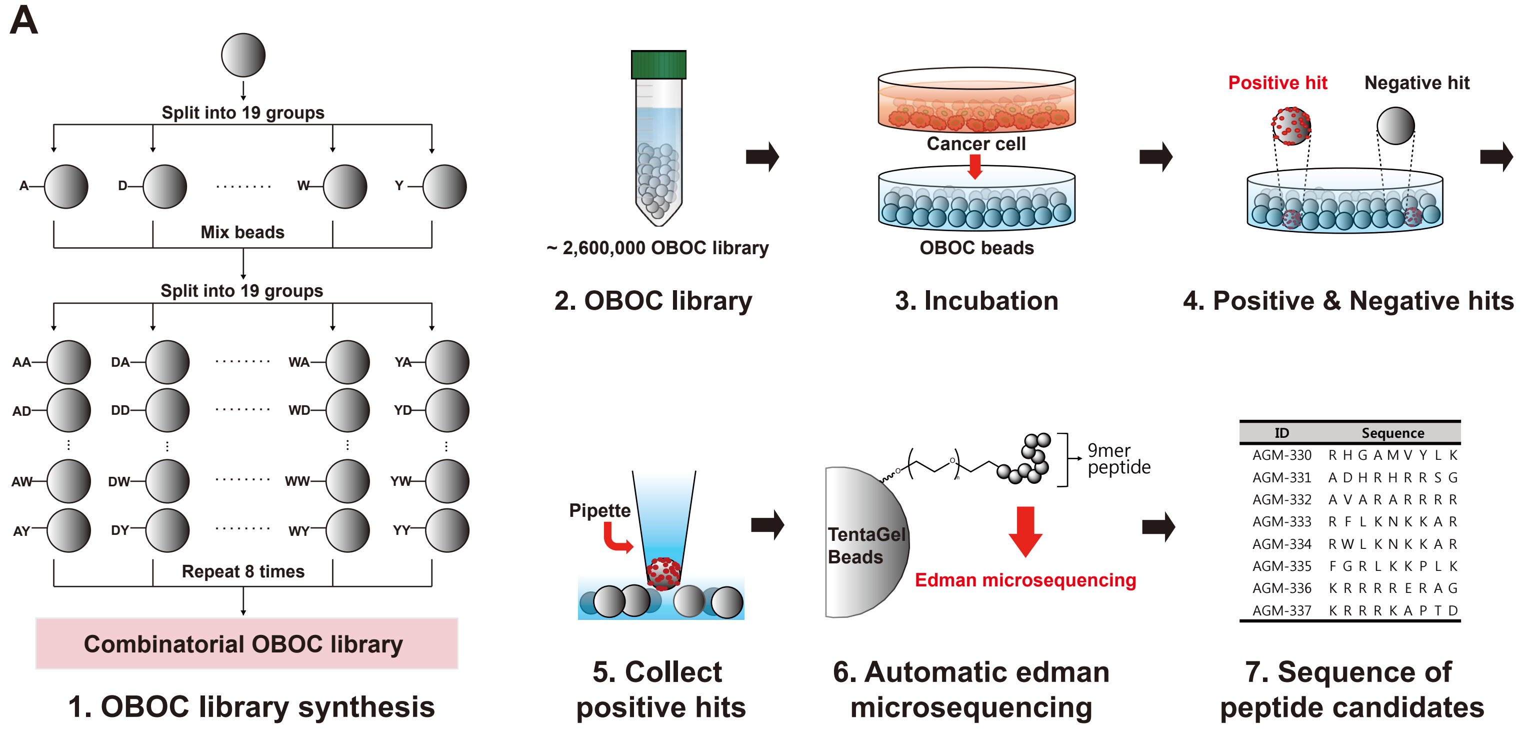

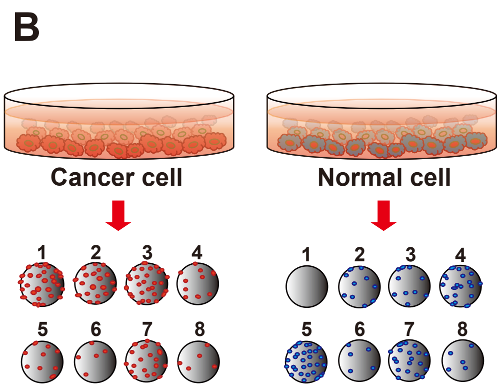

ID	Sequence	MDA-MB-231	MCF-10A
AGM-330	RHGAMVYLK	++++	-
AGM-331	ADHRHRRSG	++++	++
AGM-332	AVARARRRR	+++	++
AGM-333	RFLKNKKAR	++	+++
AGM-334	RWLKNKKAR	++	+++
AGM-335	FGRLKKPLK	+	+
AGM-336	KRRRRERAG	+++	+++
AGM-337	KRRRKAPTD	+	+

Note : Semiquantitative relative binding activity: "++++" very strong binding (>40 cells per bead), "+++" strong binding (30 -20 cells per bead), "++" moderate binding (20 - 10 cells per bead), "+" weak binding (5-10 cells per bead), "-" no binding.

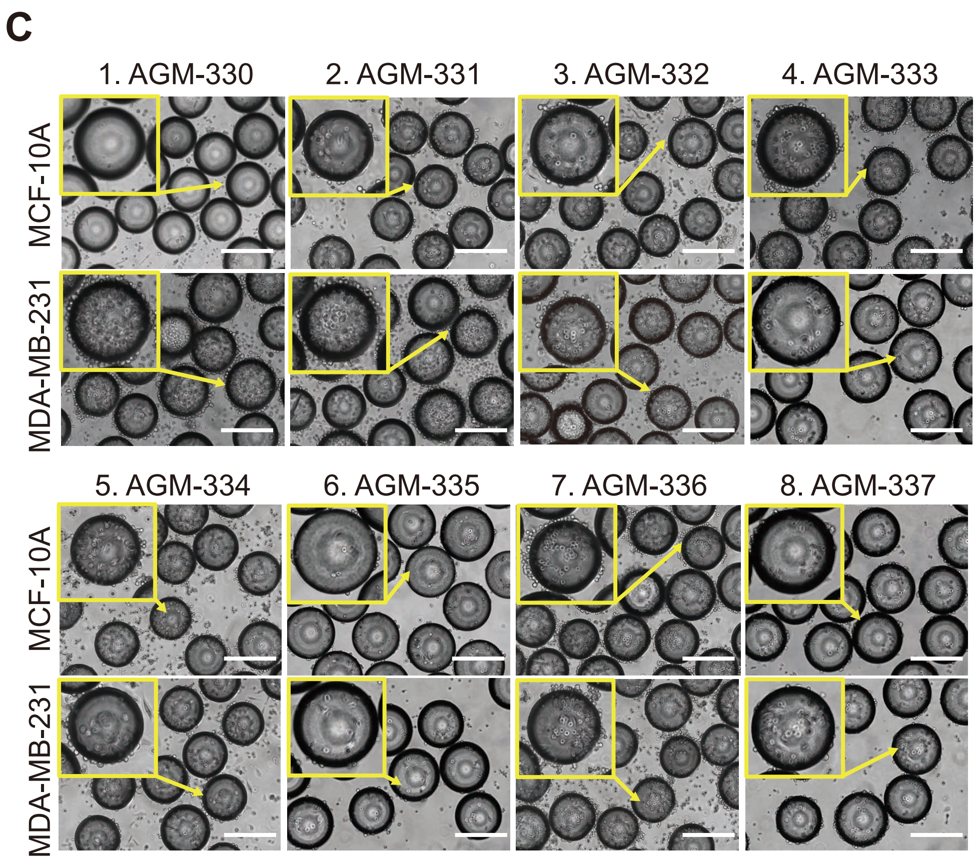

Figure S1. A, Steps performed in peptide library synthesis and screening. **1**, The split-mix synthesis method was performed to construct the combinatorial OBOC libraries. **2**, Approximately ~2,600,000 libraries were synthesized. **3**, OBOC libraries were incubated with cancer cells in a CO_2 incubator. **4**, Beads carrying ligands with an affinity for cell surface molecules became covered with cells. **5**, The positive beads were picked with a pipette under an inverted microscope. **6**, The peptide sequences for positive beads were determined by Edman microsequencing. **7**, Cancer-specific peptide ligand candidates were identified from screening OBOC combinatorial peptide libraries. **B**, The binding specificities of the peptides against cancer cell and normal cells were determined. **C**, Whole-cell binding assay results showing the cell binding specificity of 8 selected beads. Multiple breast and colorectal cancer cells and normal breast and colorectal cells were re-suspended at 10^6 cells/ml and incubated with beads. All experiments were repeated 3 times. Scale bar, 200 μm.

Figure S2

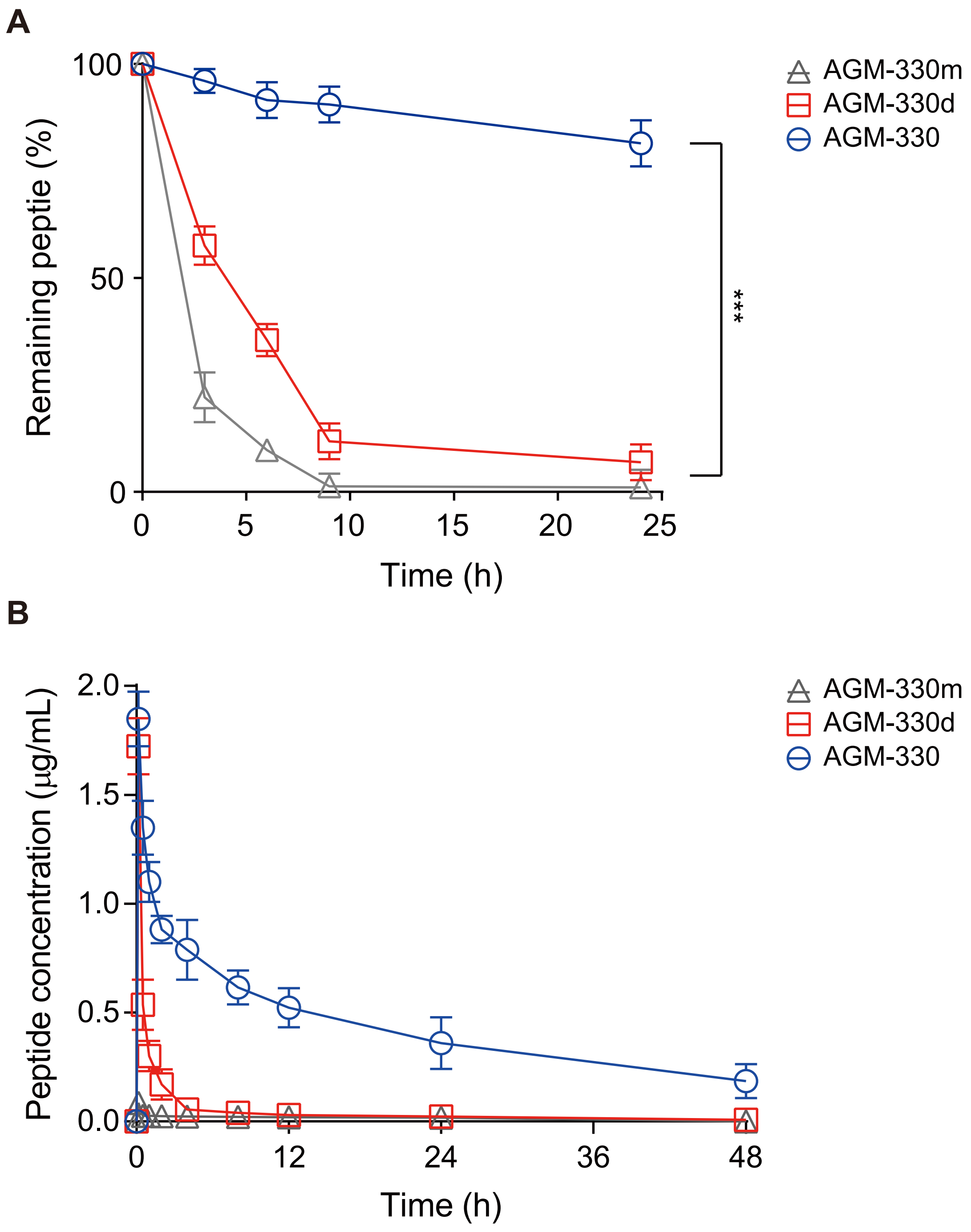

C

Parameters	AGM-330m	AGM-330d	AGM-330
**$T_{1/2}$ (h)**	0.42 ± 0.33	1.82 ± 0.36	9.43 ± 1.21
**T_{max} (h)**	0.167	0.167	0.167
**C_{max} (µg/ml)**	1.27 ± 0.12	1.71 ± 0.11	1.875 ± 0.67
**AUC (µg/h/ml)**	0.64 ± 0.09	1.77 ± 0.12	21.42 ± 0.81

$T_{1/2}$: The distribution half-time; T_{max}: Time to reach maximum plasma concentration; C_{max}: Maximum plasma concentration; AUC: Area under the plasma concentration-time curve

Figure S2. **A**, AGM-330m, AGM-330d and AGM-330 was incubated with pre-warmed human serum and incubated at 37 °C for 0, 3, 6, 9, and 24 h. The portion of serum-treated peptide remaining was determined by calculating the height of the respective elution peak in analytical-HPLC and comparing it with control without incubation. **B**, **C**, Mean plasma concentration-time profile (**B**) and pharmacokinetic parameters (**C**) of AGM-330m, AGM-330d and AGM-330 in mice ($n = 6$) after single intravenous injection. Plasma peptide concentrations were quantified by ELISA, and pharmacokinetic profiles were analyzed using Phoenix WinNonlin 8.1 (*Pharsight* Corporation, Mountain View, CA, USA). The data are presented as the means ± standard deviation. AGM-330m: Monomeric AGM-330, AGM-330d: Dimeric AGM-330, AGM-330: Tetrameric AGM-330.

Figure S3

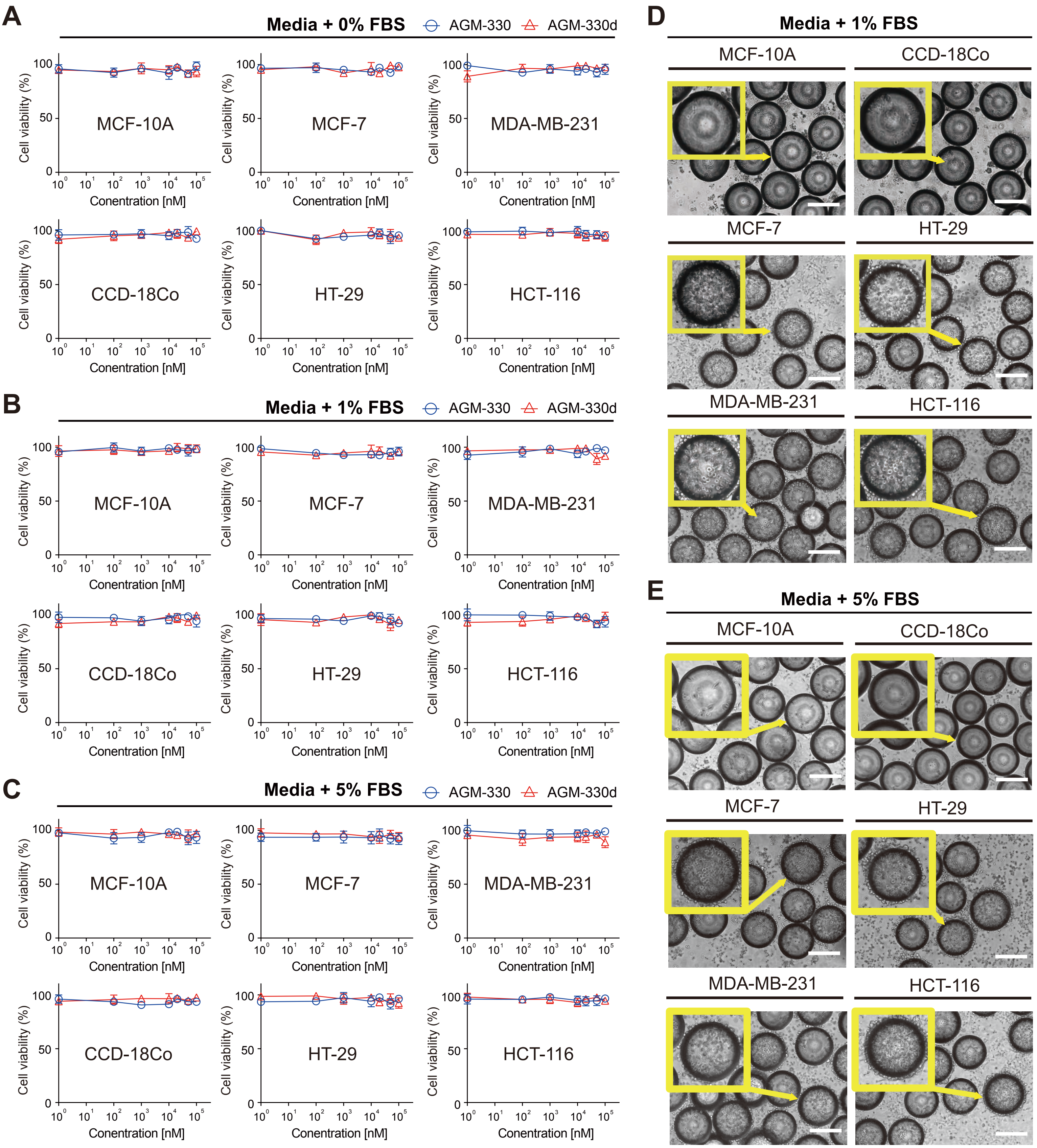

A
Media + 0% FBS
AGM-330
AGM-330d
MCF-10A
MCF-7
MDA-MB-231
CCD-18Co
HT-29
HCT-116
Cell viability (%)
0
50
100
10^0 10^1 10^2 10^3 10^4 10^5
Conentration [nM]
B
Media + 1% FBS
AGM-330
AGM-330d
MCF-10A
MCF-7
MDA-MB-231
CCD-18Co
HT-29
HCT-116
C
Media + 5% FBS
AGM-330
AGM-330d
MCF-10A
MCF-7
MDA-MB-231
CCD-18Co
HT-29
HCT-116
D
Media + 1% FBS
MCF-10A
CCD-18Co
MCF-7
HT-29
MDA-MB-231
HCT-116
E
Media + 5% FBS
MCF-10A
CCD-18Co
MCF-7
HT-29
MDA-MB-231
HCT-116

Figure S3. **A-C**, Cancer and normal cells (1×10^4 cells/well) were seeded in 96-well plates. After 24 h of incubation, the cells were treated with an increasing concentration of dimeric or tetrameric AGM-330 for 48 h. The viability of cells grown in 0 % FBS (**A**), 5 % FBS (**B**) or 10 % FBS (**C**) was assessed by a CellVia WST-1 assay (Young In Frontier, Seoul, Korea) according to the manufacturer's instruction. The numbers of viable cells were measured at a wavelength of 450 nm using an VersaMax ELISA plate reader (Molecular Devices). **D**, **E**, Binding specificity of AGM-330. A whole-cell binding assay was performed to determine the cell binding of AGM-330 in 1 % FBS (**D**) or 5 % FBS (**E**). Scale bar, 200 μm.

Figure S4

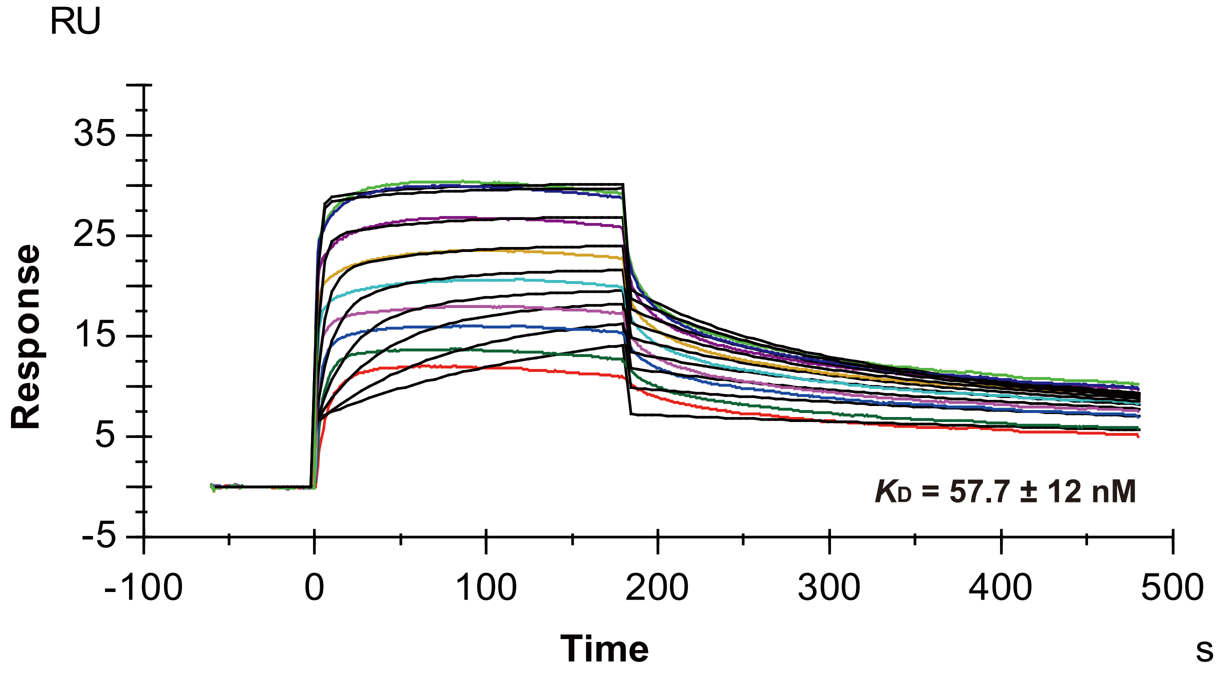

RU
Response
35
25
15
5
-5
-100
0
100
200
300
400
500
Time
s
K_D = 57.7 ± 12 nM

Figure S4. AGM-330 affinity for recombinant NCL was assessed by SPR using an increasing concentration (20 nM - 2.5 μM) of AGM-330. The SPR sensorgrams shown are from an experiment that is representative of a set of three separate experiments using different sensor chips with similar results. Recombinant NCL was immobilized onto a CM5 sensorchip. Different concentrations of AGM-330 were incubated with immobilized NCL and analyzed on a Biacore T-200 apparatus. The resulting K_D values are also indicated.

Figure S5

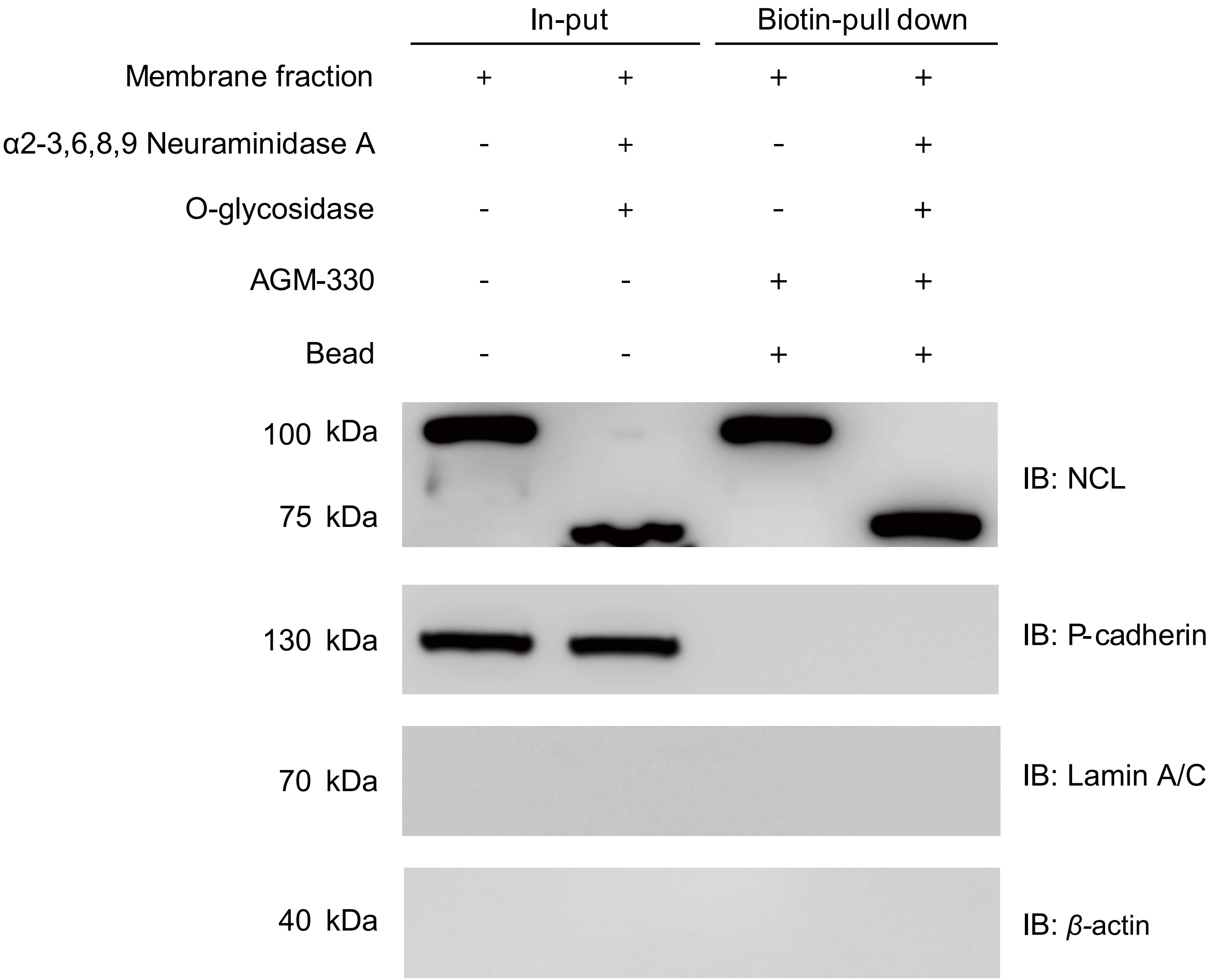
In-put
Biotin-pull down
Membrane fraction + + + +
α2-3,6,8,9 Neuraminidase A - + - +
O-glycosidase - + - +
AGM-330 - - + +
Bead - - + +
100 kDa
75 kDa
IB: NCL
130 kDa
IB: P-cadherin
70 kDa
IB: Lamin A/C
40 kDa
IB: β-actin

Figure S5. Analysis of the interaction between AGM-330 and *O*-glycan-cleaved membrane NCL by a biotin pull-down assay. Immunoblotting analysis of the eluted proteins from the biotin pull-down using an anti-NCL antibody. Lane 1: Input of membrane NCL; Lane 2: Input of *O*-glycan-cleaved membrane NCL; Lane 3: Elution after incubation with membrane NCL; Lane 4: Elution after incubation with *O*-glycan-cleaved membrane NCL.

Figure S6

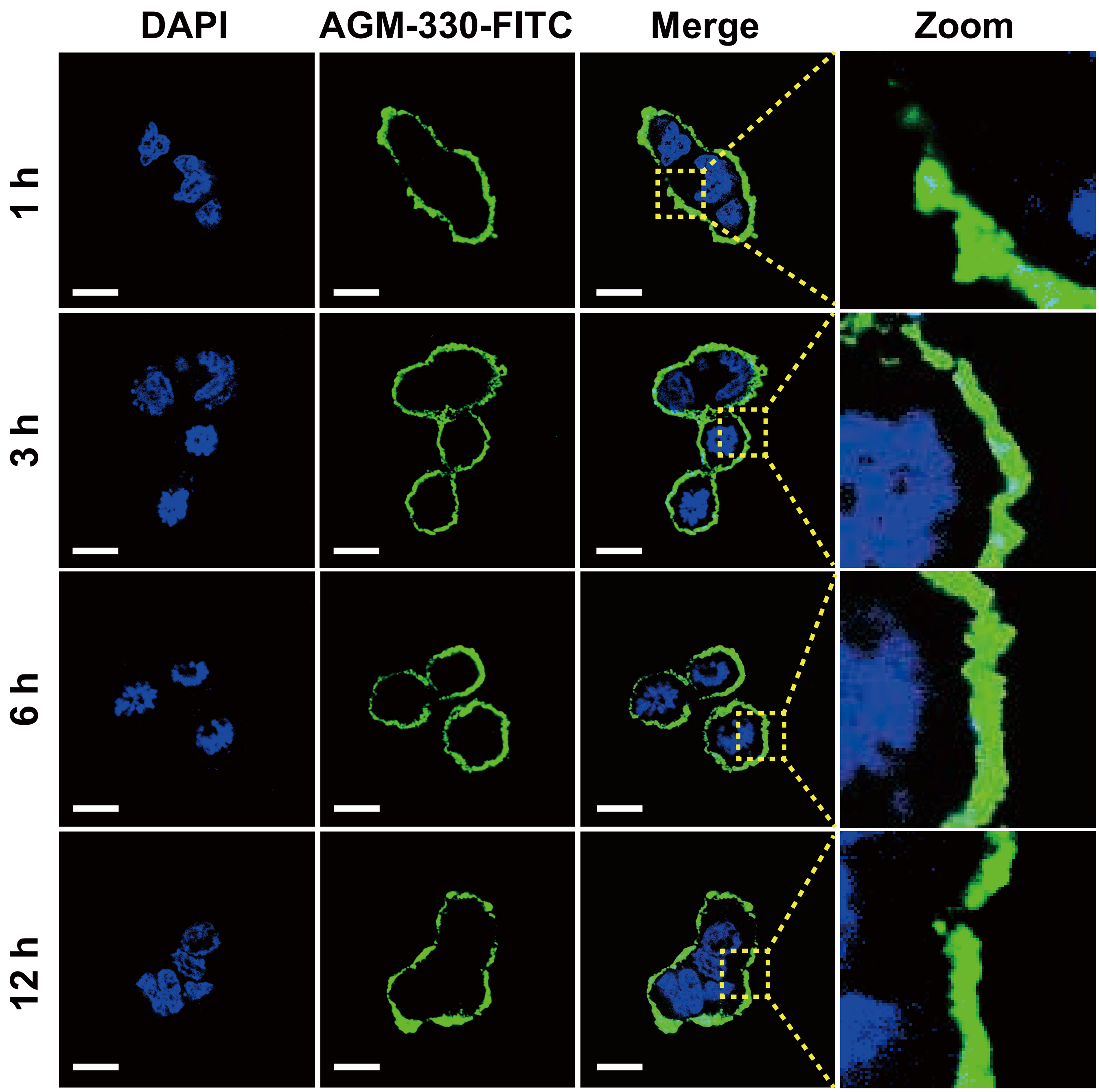

Figure S6. Time-dependent *in vitro* binding assay of AGM-330-FITC toward cancer cells. MDA-MB-231 cells were incubated with 5 μmol/l AGM-330-FITC for 1, 3, 6 and 12 h at 37 °C. The cells were then fixed with 4 % paraformaldehyde, and the nuclei were stained with DAPI. MERGE represents a merged image of AGM-330-FITC and nuclear staining by DAPI. AGM-330-FITC: FITC-conjugated AGM-330. Scale bar, 20 μ m.

Figure S7

A

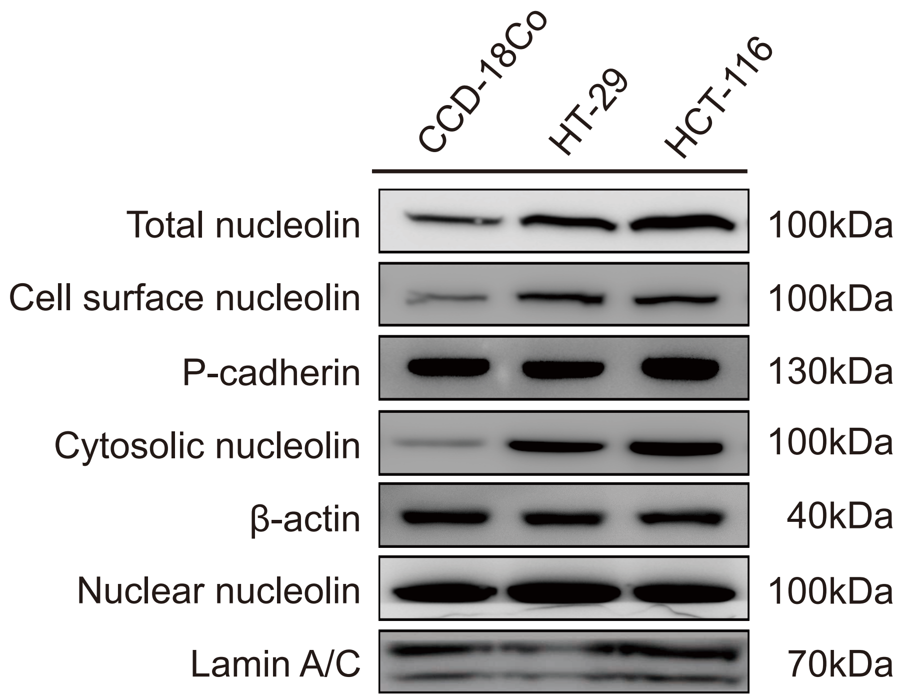
CCD-18Co
HT-29
HCT-116
Total nucleolin
Cell surface nucleolin
P-cadherin
Cytosolic nucleolin
β-actin
Nuclear nucleolin
Lamin A/C
100kDa
100kDa
130kDa
100kDa
40kDa
100kDa
70kDa

B

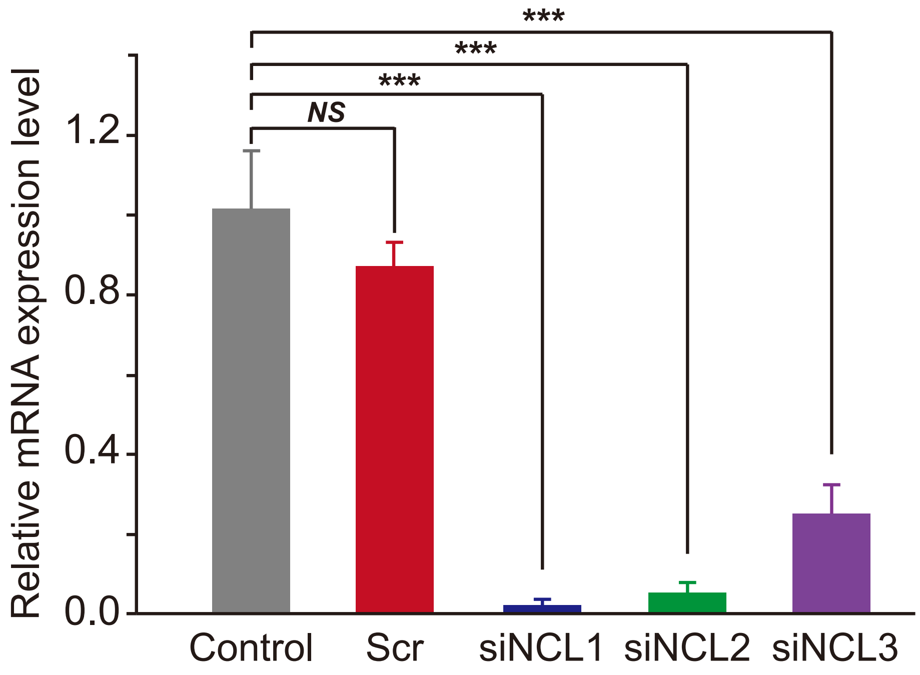
Relative mRNA expression level
1.2
0.8
0.4
0.0
***
***
***
NS
Control
Scr
siNCL1
siNCL2
siNCL3

Figure S7

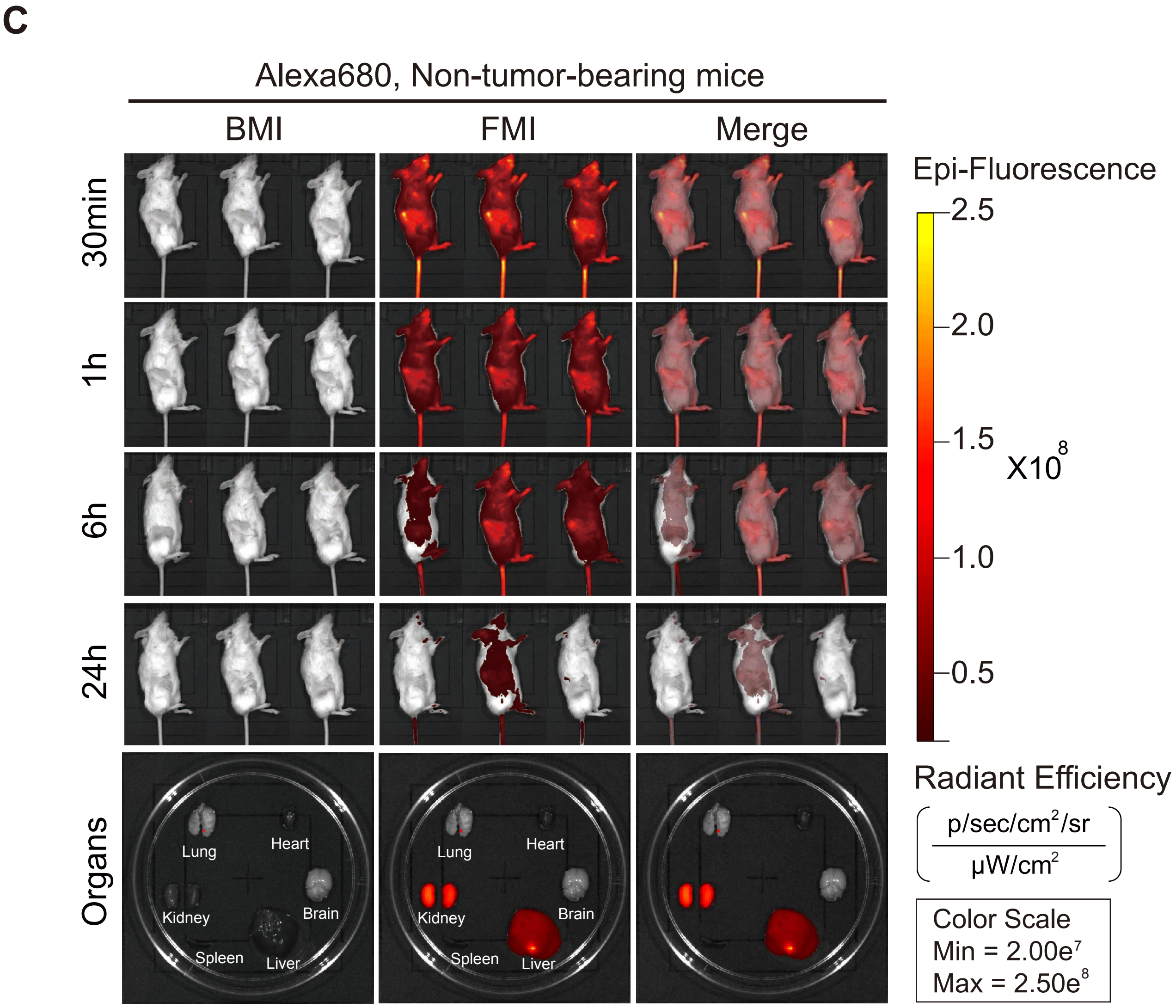
C
Alexa680, Non-tumor-bearing mice
BMI
FMI
Merge
30min
1h
6h
24h
Organs
Lung
Heart
Kidney
Brain
Spleen
Liver
Epi-Fluorescence
2.5
2.0
1.5
1.0
0.5
X10^8
Radiant Efficiency
p/sec/cm²/sr
μW/cm²
Color Scale
Min = 2.00e7
Max = 2.50e8

Figure S7. **A**, The NCL distribution in various subcellular fractions was analyzed in CCD-18Co, HT-29, and HCT-116 cells. Plasma membrane, cytosol and nuclear NCL were immunoblotted using an anti-NCL antibody. **B**, MDA-MB-231 cells were transfected with non-targeting scrambled siRNA (Scr) or NCL-targeting siNCL1, siNCL2, or siNCL3. After 24 h, the efficiencies of gene silencing were determined by real-time PCR. Based on the observed mRNA levels, one set of siRNAs (siNCL1) showed significant target gene knockdown efficiency and was used in further experiments. **C**, *In vivo* fluorescence images of mice 30 min, 1 h, 6 h, and 24 h after intravenous injections of 10 nmol free Alexa680 in non-tumor-bearing mice. Fluorescence images of the major organs, including the heart, spleen, lung, brain, liver, kidney, and tumors removed from mice with different treatments. The bar graphs show the means ± SD, and statistical analyses were performed using one-way ANOVA with Dunnett's multiple comparison. *, **, and *** indicate $P < 0.05$, $P < 0.01$, and $P < 0.001$, respectively.

Figure S8

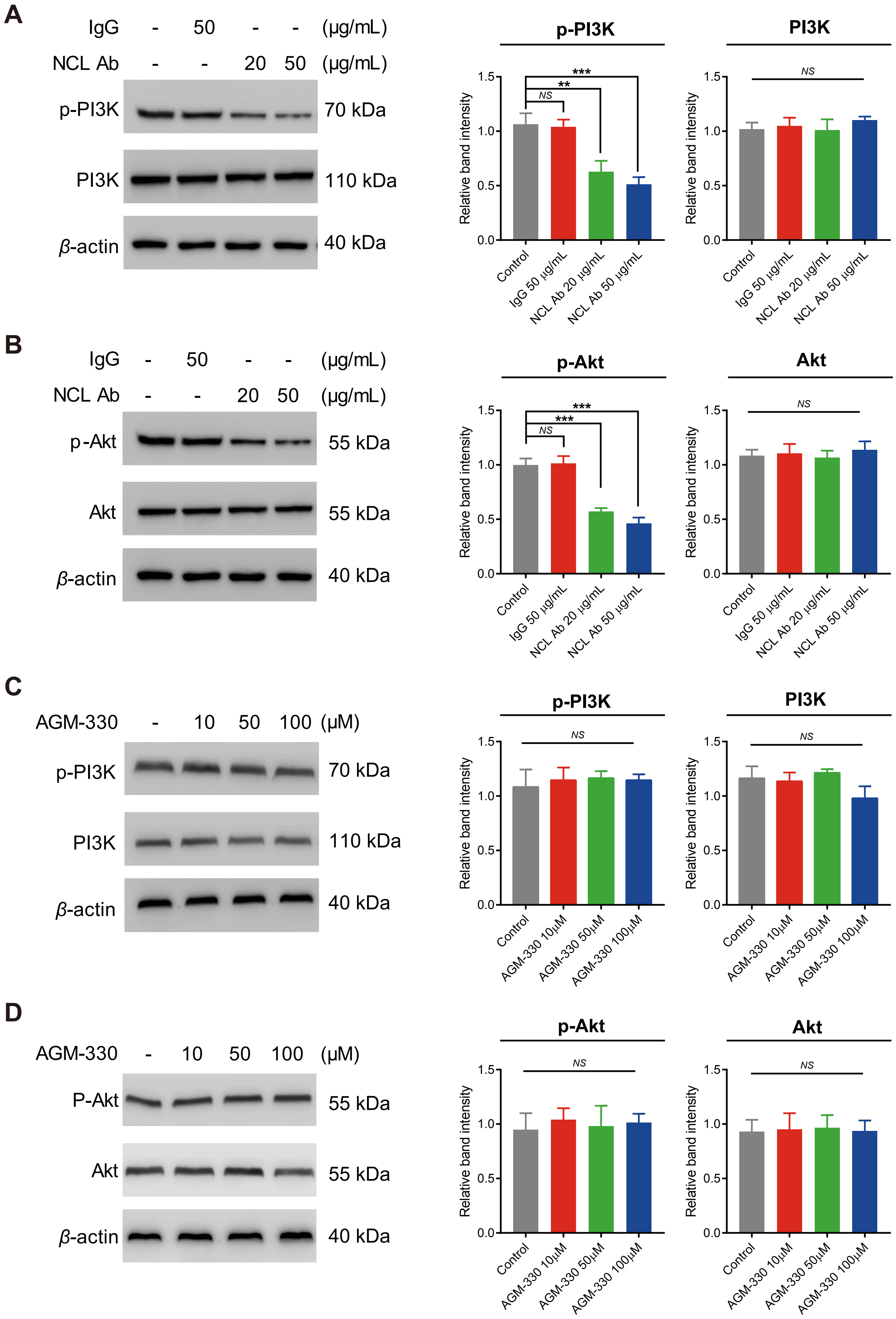

A
IgG - 50 - - (μg/mL)
NCL Ab - - 20 50 (μg/mL)
p-PI3K 70 kDa
PI3K 110 kDa
β-actin 40 kDa
p-PI3K
PI3K
Relative band intensity
NS
**
***
Control
IgG 50 μg/mL
NCL Ab 20 μg/mL
NCL Ab 50 μg/mL
B
IgG - 50 - - (μg/mL)
NCL Ab - - 20 50 (μg/mL)
p-Akt 55 kDa
Akt 55 kDa
β-actin 40 kDa
p-Akt
Akt
C
AGM-330 - 10 50 100 (μM)
p-PI3K 70 kDa
PI3K 110 kDa
β-actin 40 kDa
Control
AGM-330 10μM
AGM-330 50μM
AGM-330 100μM
D
AGM-330 - 10 50 100 (μM)
P-Akt 55 kDa
Akt 55 kDa
β-actin 40 kDa

Figure S8. **A-D**, Western blot analysis of PI3K/Akt signaling in MDA-MB-231 cells stimulated by increasing concentrations of an anti-NCL antibody and AGM-330. After treatment with the anti-NCL antibody (**A**,**B**) or AGM-330 (**C**,**D**), the protein levels of p-PI3K, PI3K, p-Akt and Akt were assessed by immunoblot analysis. The bar graphs show the means ± SD, and statistical analyses were performed using one-way ANOVA with Dunnett's multiple comparison. *, **, and *** indicate $P < 0.05$, $P < 0.01$, and $P < 0.001$, respectively.

Figure S9

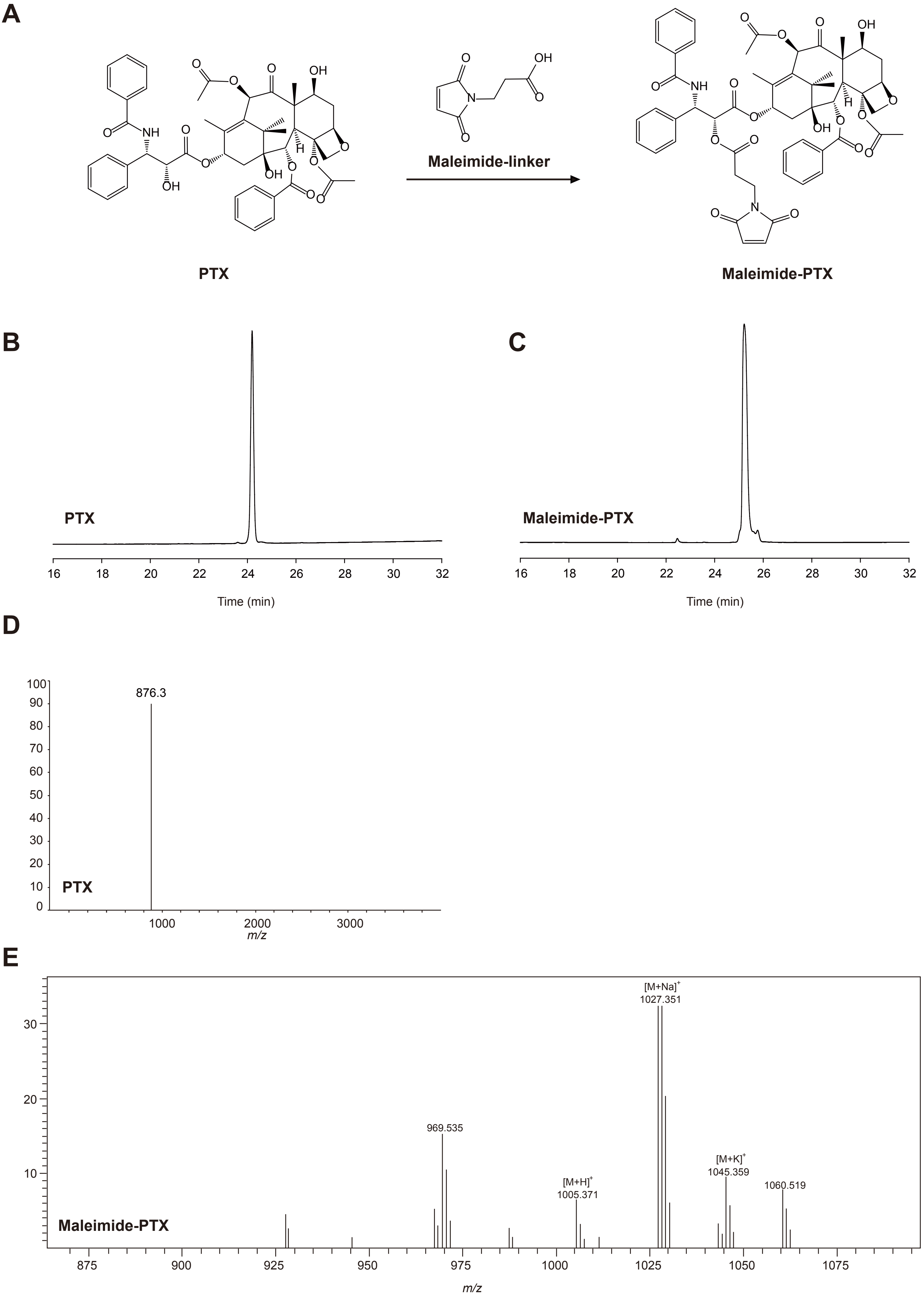
A
PTX
Maleimide-linker
Maleimide-PTX
B
PTX
Time (min)
C
Maleimide-PTX
Time (min)
D
876.3
PTX
m/z
E
[M+Na]+
1027.351
969.535
[M+H]+
1005.371
[M+K]+
1045.359
1060.519
Maleimide-PTX
m/z

Figure S9

F

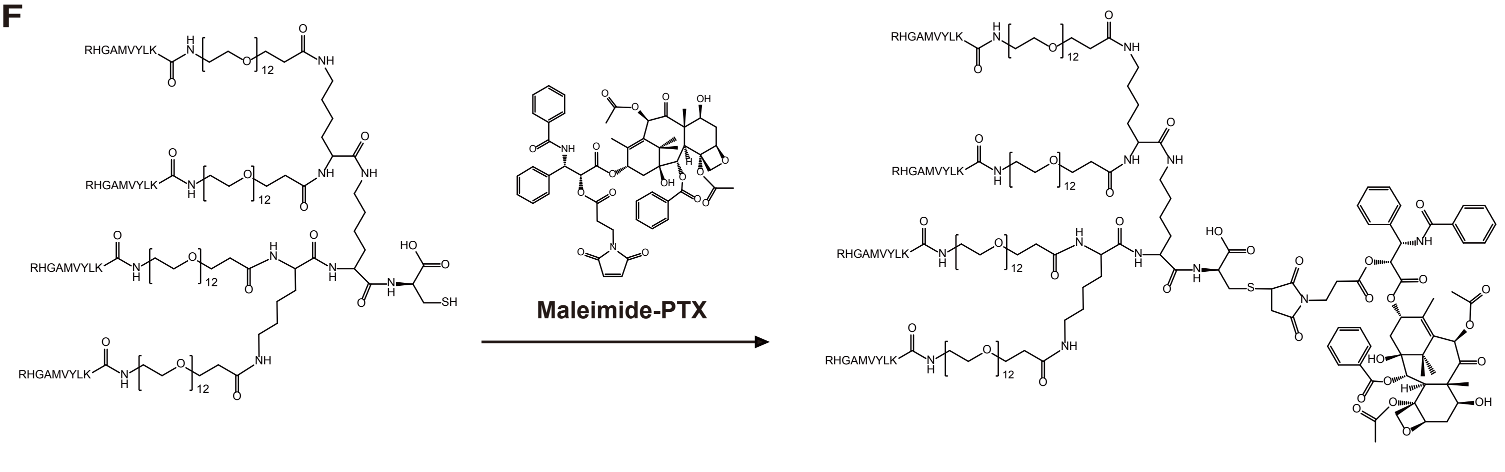

AGM-330 **AGM-330-PTX**

G

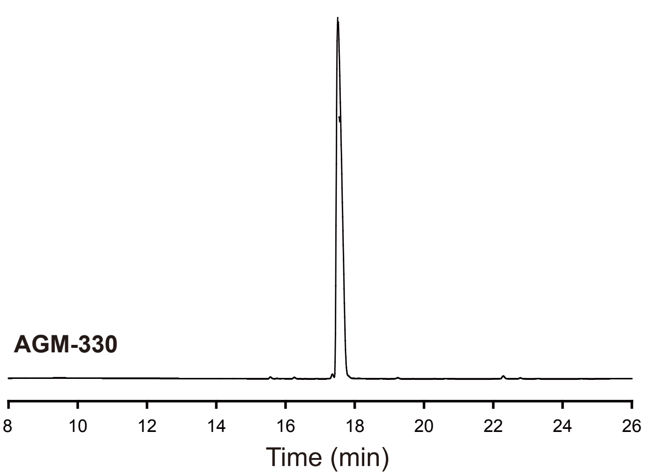

H

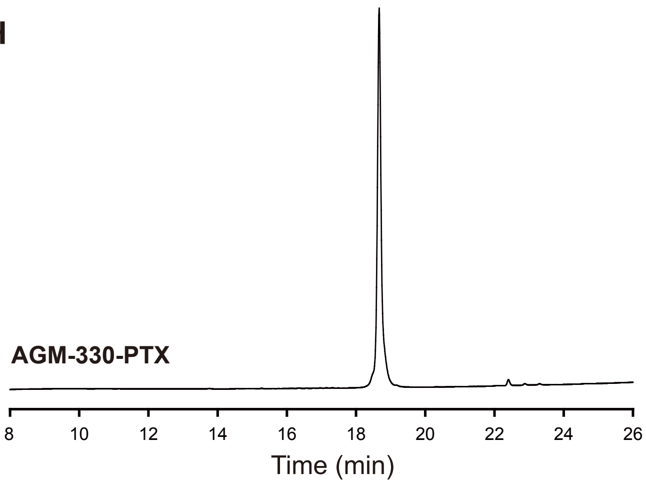

I

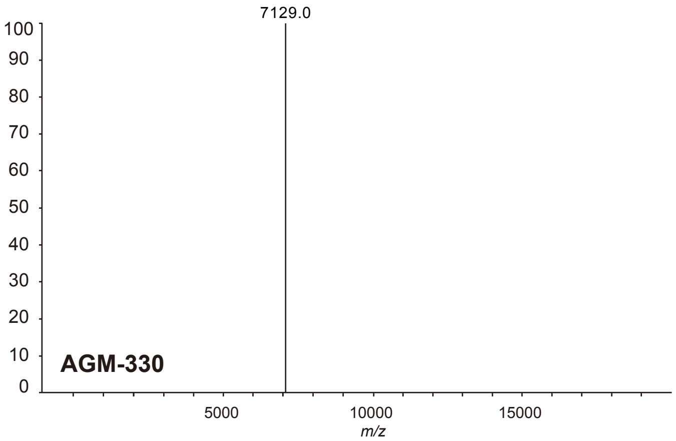

J

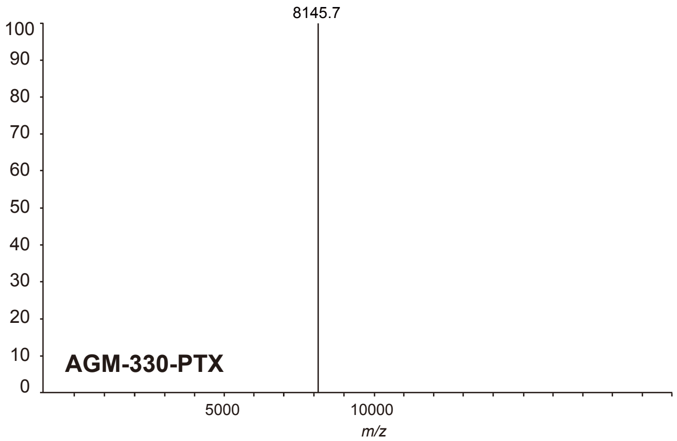

Figure S9

K

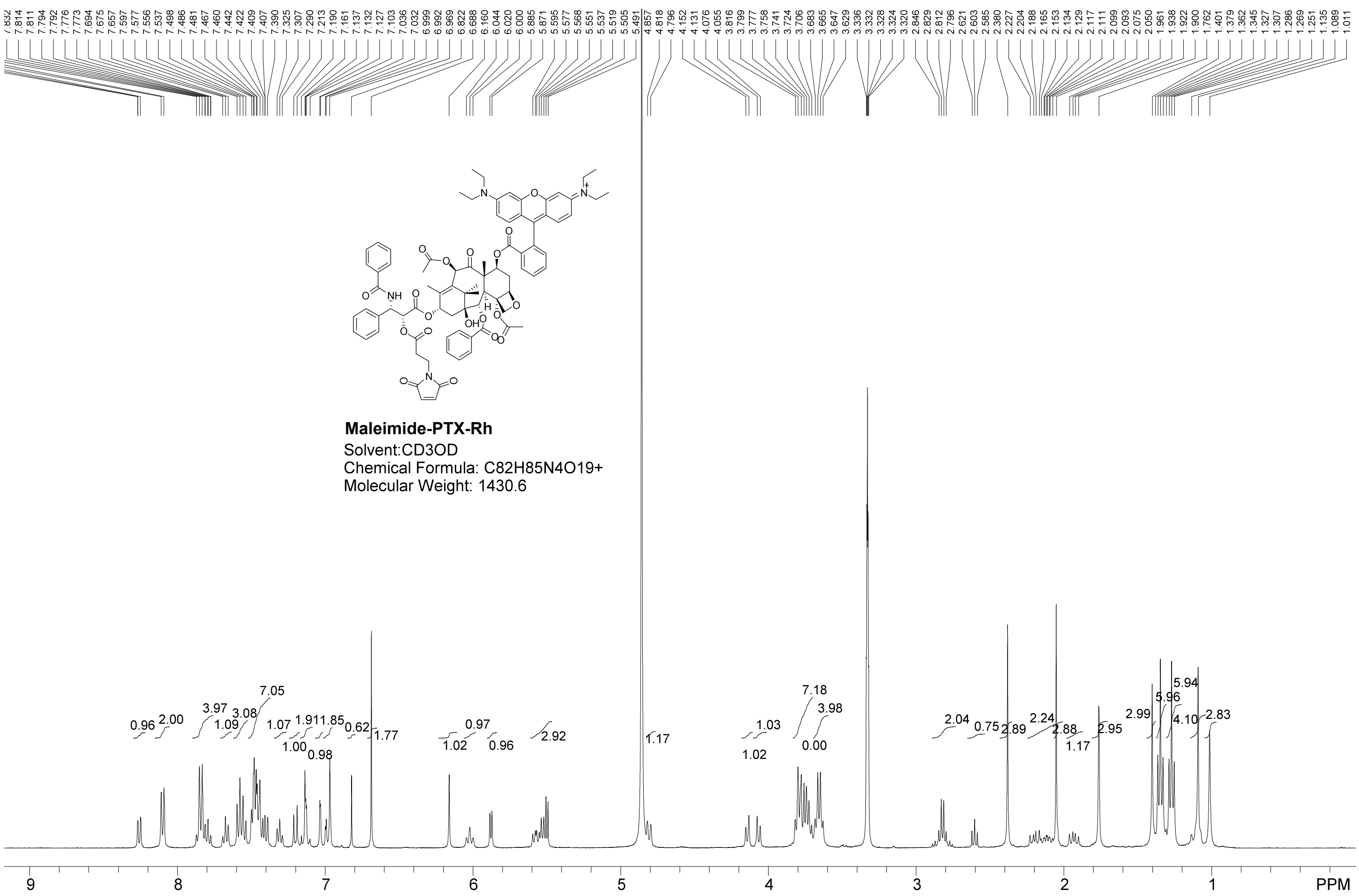

L

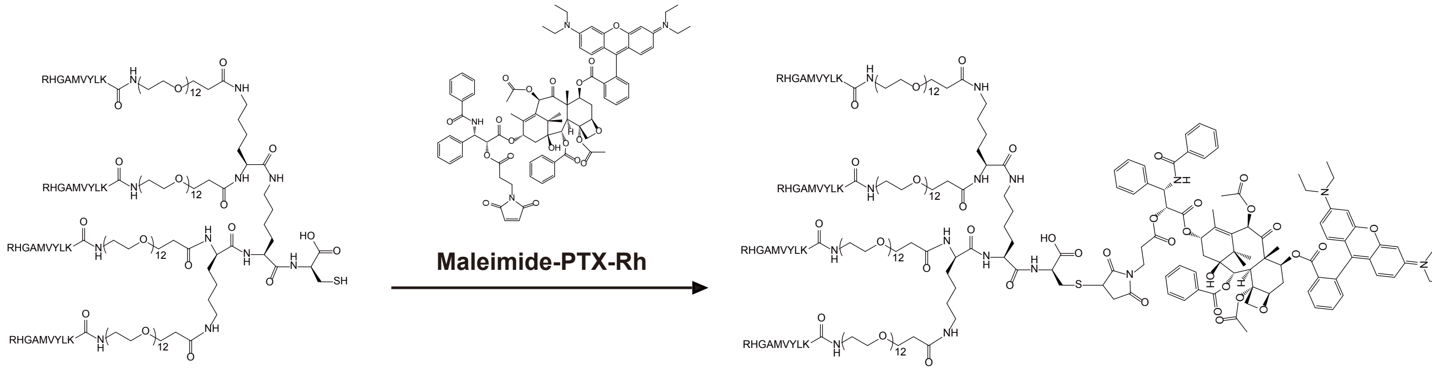

AGM-330

AGM-330-PTX-Rh

M

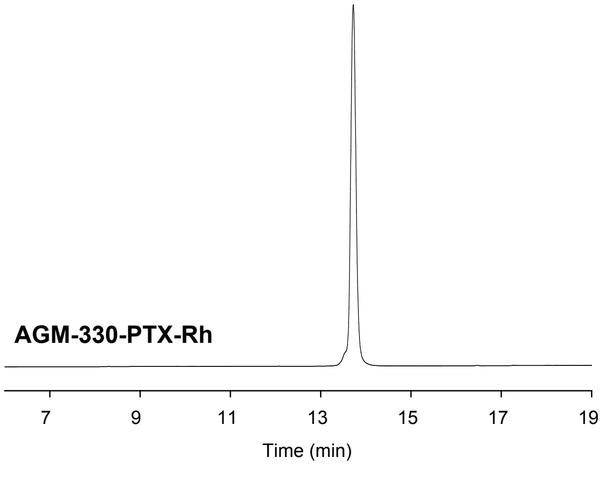

N

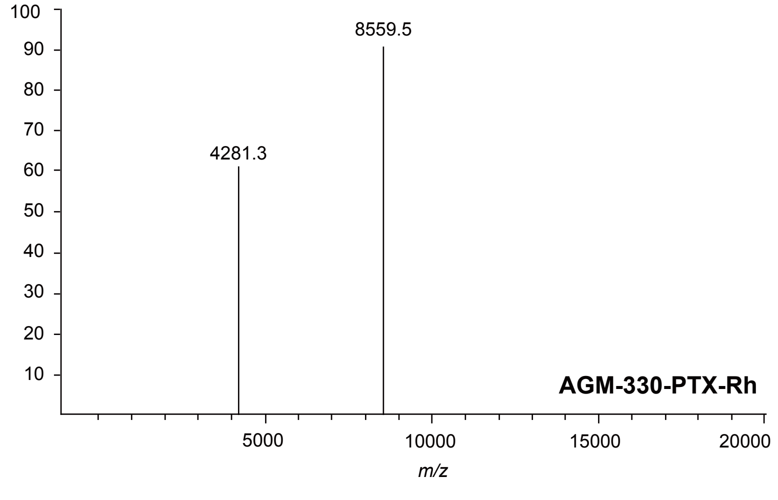

Figure S9. **A**, Conjugation of paclitaxel (PTX) with a maleimide linker. **B-E**, HPLC chromatograms and MS spectra of PTX and maleimide-PTX. The HPLC chromatograms of PTX (**B**) and maleimide-PTX (**C**). MS spectra of PTX (**D**) by MALDI-TOF and maleimide-PTX (**E**) by ESI-MS. **F**, Conjugation of AGM-330 with maleimide-PTX. **G-J**, HPLC chromatograms and MS spectra of AGM-330 and AGM-330-PTX. The HPLC chromatograms of AGM-330 (**G**) and AGM-330-PTX (**H**). MS spectra of AGM-330 (**I**) and AGM-330-PTX (**J**) by MALDI-TOF. **K**, ^{1}H-NMR spectra of maleimide-PTX- Rh. ^{1}H-NMR: (400 MHz, CD3OD): δ 8.27 (d, *J* = 0.8Hz, 1H), 8.25-8.11 (m, 2H), 8.09-7.77 (m, 4H),7.69-7.65 (m, 1H), 7.59-7,53 (m, 3H), 7.49-7.39 (m, 7H), 7.31 (t, *J* =7.2Hz, 1H),7.20 (d, *J* =9.2Hz ,1H), 7.13-7.10 (m, 2H), 7.03(d, *J* = 0.8Hz, 1H), 6.99-6.95 (m , 2H) ,6.82-6.68 (m, 2H), 6.16 (s, 1H), 6.02 (t, *J* = 8Hz, 1H), 5.87 (d, *J* = 6Hz, 1H), 5.59-5.49 (m, 3H), 4.80 (d, *J* = 8.8Hz, 1H), 4.14 (d, *J* = 8.4Hz, 1H) , 4.06 (d, *J* = 8.4Hz, 1H), 3.82-3.70 (m, 7H), 3.68-3.63 (m, 4H), 2.84-2.79 (m, 2H), 2.60 (t, *J* = 7.2Hz, 1H) ,2.38(s, 3H), 2.22-2.07 (m, 2H), 2.05 (s, 3H) 1.96-1.90 (m, 1H), 1.76 (s, 3H) , 1.40 (s, 3H), 1.37-1.25(m, 12H), 1.13-1.08 (m, 4H), 1.01 (s, 3H). **L**, Conjugation of AGM-330 with maleimide-PTX-Rh. **M**, **N**, HPLC chromatogram (**M**) and MS spectrum (**N**) of AGM-330-PTX-Rh. Maleimide-PTX: maleimide-linker conjugated PTX; AGM-330-PTX: maleimide-PTX conjugated AGM-330; maleimide-PTX-Rh: rhodamine B conjugated maleimide-PTX; AGM-330-PTX-Rh: maleimide-PTX-Rh conjugated AGM-330.

Figure S10

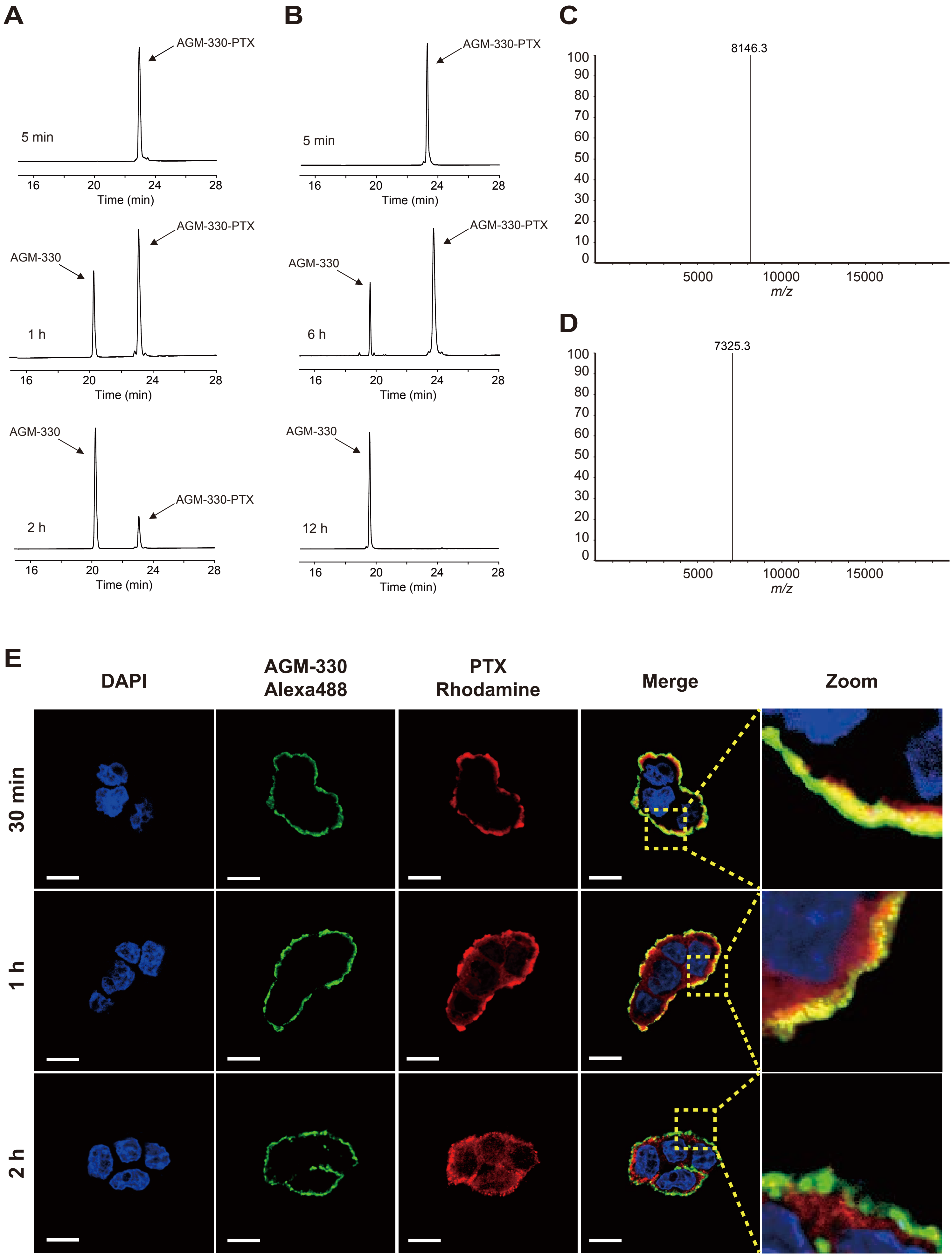
A
AGM-330-PTX
5 min
16
20
24
28
Time (min)
AGM-330
AGM-330-PTX
1 h
2 h
B
6 h
12 h
C
8146.3
100
90
80
70
60
50
40
30
20
10
0
5000
10000
15000
m/z
D
7325.3
E
DAPI
AGM-330
Alexa488
PTX
Rhodamine
Merge
Zoom
30 min
1 h
2 h

Figure S10. **A**, **B**, Changes in the HPLC chromatogram of AGM-330-PTX after incubation with human serum (**A**) or PBS (**B**). **C**, **D**, MS spectra of AGM-330-PTX (**C**) and AGM-330 (**D**). **E**, Time-dependent fluorescence imaging of AGM-330-PTX-Rh in MDA-MB-231 cells. Cells were incubated with 5 μmol/l AGM-330-PTX-Rh. AGM-330 was stained with a primary anti-AGM-330 antibody, and the complex was revealed using an anti-rabbit secondary antibody coupled to Alexa Fluor 488. Cells were then fixed with 4 % paraformaldehyde, and the nuclei were stained with DAPI. MERGE represents a merged image of AGM-330, PTX, and nuclear staining by DAPI. Scale bar, 20 μm.

Figure S11

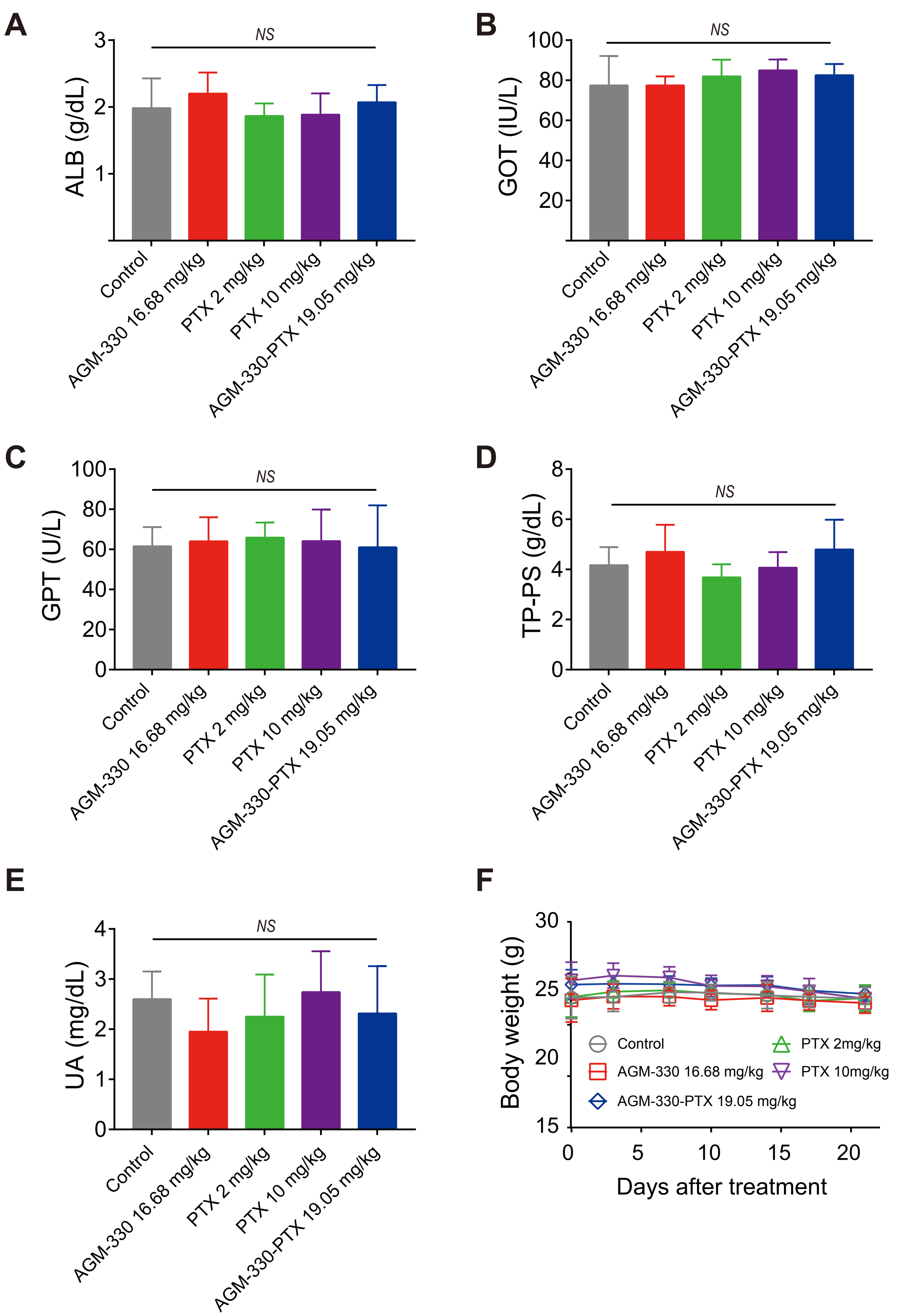

A
ALB (g/dL)
NS
B
GOT (IU/L)
NS
C
GPT (U/L)
NS
D
TP-PS (g/dL)
NS
E
UA (mg/dL)
NS
Control
AGM-330 16.68 mg/kg
PTX 2 mg/kg
PTX 10 mg/kg
AGM-330-PTX 19.05 mg/kg
F
Body weight (g)
Days after treatment
Control
PTX 2mg/kg
AGM-330 16.68 mg/kg
PTX 10mg/kg
AGM-330-PTX 19.05 mg/kg

Figure S11. **A-E**, NPG™ mice bearing MDA-MB-231-luc tumors were treated twice a week for 21 days with intravenous doses of vehicle (PBS), PTX (2 or 10 mg/kg), AGM-330 (16.68 mg/kg, molar equivalent to 2 mg/kg PTX) and AGM-330-PTX (19.05 mg/kg, molar equivalent to 2 mg/kg PTX) (n = 5). Blood was collected from xenografted mice under isoflurane-induced deep anesthesia by cardiac puncture. After allowing for blood coagulation at 4 °C, serum was collected by centrifugation at 3000 rpm for 10 min at 4 °C. Analysis of the levels of (**A**), ALB, (**B**), GOT, (**C**), GPT, (**D**), TP-PS, and (**E**), UA in the serum was performed using a veterinary hematology analyzer (Fuji DRI-Chem 3500 s, Fujifilm, Tokyo, Japan) according to the manufacturer's provided protocols. ALB, albumin; GOT, glutamic oxaloacetic transaminase; GTP, glutamate pyruvate transaminase; TP-PS, total protein/protein scan; UA, uric acid. **F**, Body weight changes after 3 weeks of different treatments indicated in xenograft mice. AGM-330d-PTX: PTX-conjugated dimeric AGM-330. The bar graphs show the means ± SD, and statistical analyses were performed using one-way ANOVA with Dunnett's multiple comparison. *, **, and *** indicate $P < 0.05$, $P < 0.01$, and $P < 0.001$, respectively.A

Figure S12

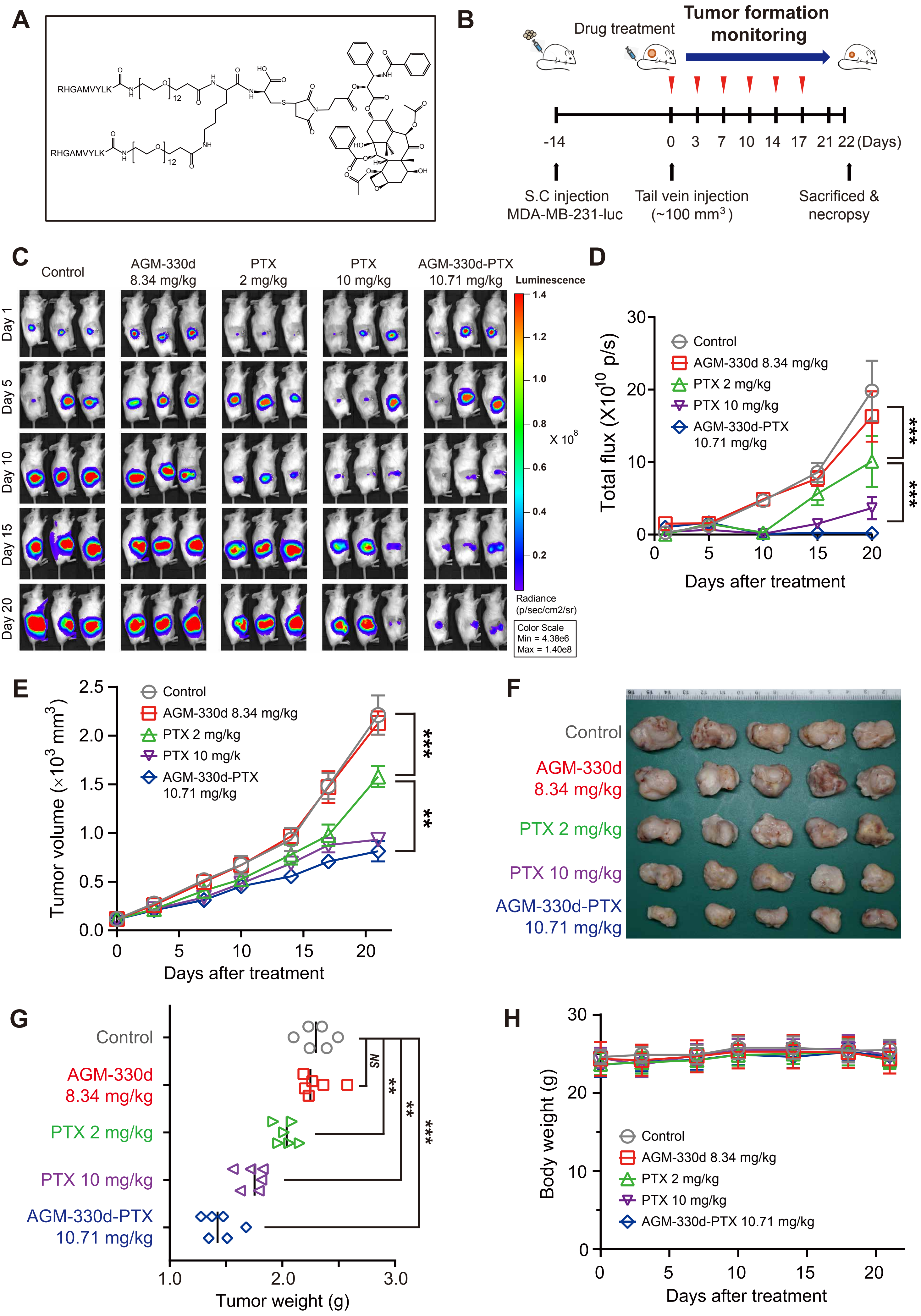
A
RHGAMVYLK
RHGAMVYLK
B
Drug treatment
Tumor formation monitoring
-14 0 3 7 10 14 17 21 22 (Days)
S.C injection MDA-MB-231-luc
Tail vein injection (~100 mm3)
Sacrificed & necropsy
C
Control
AGM-330d 8.34 mg/kg
PTX 2 mg/kg
PTX 10 mg/kg
AGM-330d-PTX 10.71 mg/kg
Luminescence
Day 1
Day 5
Day 10
Day 15
Day 20
X 10^8
Radiance (p/sec/cm2/sr)
Color Scale Min = 4.38e6 Max = 1.40e8
D
Total flux (X10^10 p/s)
Days after treatment
Control
AGM-330d 8.34 mg/kg
PTX 2 mg/kg
PTX 10 mg/kg
AGM-330d-PTX 10.71 mg/kg
E
Tumor volume (×10^3 mm^3)
Days after treatment
Control
AGM-330d 8.34 mg/kg
PTX 2 mg/kg
PTX 10 mg/k
AGM-330d-PTX 10.71 mg/kg
F
Control
AGM-330d 8.34 mg/kg
PTX 2 mg/kg
PTX 10 mg/kg
AGM-330d-PTX 10.71 mg/kg
G
Control
AGM-330d 8.34 mg/kg
PTX 2 mg/kg
PTX 10 mg/kg
AGM-330d-PTX 10.71 mg/kg
NS
Tumor weight (g)
H
Body weight (g)
Days after treatment
Control
AGM-330d 8.34 mg/kg
PTX 2 mg/kg
PTX 10 mg/kg
AGM-330d-PTX 10.71 mg/kg

Figure S12. **A**, Structure of AGM-330d-PTX. **B**, Schematic representation of the experimental protocol as described in the Materials and Methods section. Anesthetized 6 week-old male NPG mice were inoculated with a 1:1 mix of Matrigel and 1 × 10^6 MDA-MB-231-luc cells into the mammary fat pad. When the volume of the primary tumors reached approximately 100 mm^3, tumor-bearing mice were treated with vehicle (PBS), PTX (2 or 10 mg/kg), AGM-330d (8.34 mg/kg, molar equivalent to 2 mg/kg PTX), or AGM-330d-PTX (10.71 mg/kg, molar equivalent to 2 mg/kg PTX) (n = 5/group). **C**, Monitoring of tumor growth through whole-body bioluminescence imaging. The mice were subjected to bioluminescence imaging every 5 days. **D**, Bioluminescence was quantified for each time point using the region of interest tool in the Living Image software program. **E-G**, The therapeutic effect of AGM-330d-PTX was evaluated in breast cancer xenograft models. MDA-MB-231-luc cells were subcutaneously inoculated into the mammary fat pads of NPG mice. (**E**), The primary tumor volume was measured twice per week until the day of sacrifice. The primary tumor volume was calculated using the following formula: volume (mm^3) = [length (mm)] × [width (mm)]^2 × 0.5. (**F**), On the day of sacrifice, all primary tumors were isolated, and (**G**) the primary tumor weights were evaluated. **H**, Body weight changes after 3 weeks of different treatments in the indicated xenograft mice. AGM-330d-PTX: PTX-conjugated dimeric AGM-330. Bar graphs represent the means ± SD, and statistical analyses were performed using one-way ANOVA with Dunnett's multiple comparison. *, **, and *** indicate $P < 0.05$, $P < 0.01$, and $P < 0.001$, respectively.
